# Noble gas electrochemistry-driven luminescence integrated with machine learning for environmental monitoring

**DOI:** 10.1039/d6sc04632k

**Published:** 2026-10-09

**Authors:** Ziyu Wang, Wenxuan Zhao, Qiao Sun, Yulin Li, Chengqi Li, Meiyun Xu, Jing-Juan Xu, Daoben Hua

**Affiliations:** a Jiangsu Key Laboratory of Radiation Injury Prevention and Hazardous Substance Control, School for Radiological and Interdisciplinary Sciences (RAD-X), Collaborative Innovation Center of Radiological Medicine of Jiangsu Higher Education Institutions, Soochow University 199 Ren'ai Road Suzhou 215123 P. R. China dbhua_lab@suda.edu.cn; b State Key Laboratory of Analytical Chemistry for Life Science, School of Chemistry and Chemical Engineering, Nanjing University Nanjing 210023 P. R. China xujj@nju.edu.cn

## Abstract

Noble gases are traditionally known as not being easily involved in chemical reactions owing to their stable electronic configuration. Here, we study the electrochemical redox and electrochemiluminescence (ECL) mechanisms of a series of noble gases (radon, xenon and krypton) for the first time and demonstrate their ability to produce an ECL signal with an emission peak in the range of 600–640 nm. Among them, the key intermediates Rn˙^+^/Xe˙^+^ are observed *via* HPLC-MS to confirm their electrochemical redox and ECL process. Additionally, the anodic/cathodic ECL behavior and the ECL intensity of radon in an annihilation state are confirmed to depend on the interaction between solvent molecules and Rn˙^−^/Rn˙^+^, which can be summarized as a free radical-solvent interaction-dependent ECL (FSDE) mechanism. A machine learning framework with SHapley additive exPlanations analysis is also integrated to enable high-fidelity differentiation of Rn/Xe for accurate determination. This work advances our knowledge of the electrochemistry of noble gases and develops a machine-learning-assisted trace radon/xenon monitoring method with high selectivity and sensitivity. The ECL behavior is successfully employed for noble gas determination in various real-world atmospheric samples, indicating its application potential in the fields of environment, geoscience, and public health.

## Abstract 2

The electrochemical redox and electrochemiluminescence of noble gases are studied for the first time, which advances our knowledge of the electrochemistry of noble gases and can be applied in trace radon/xenon monitoring.

## Graphical abstract

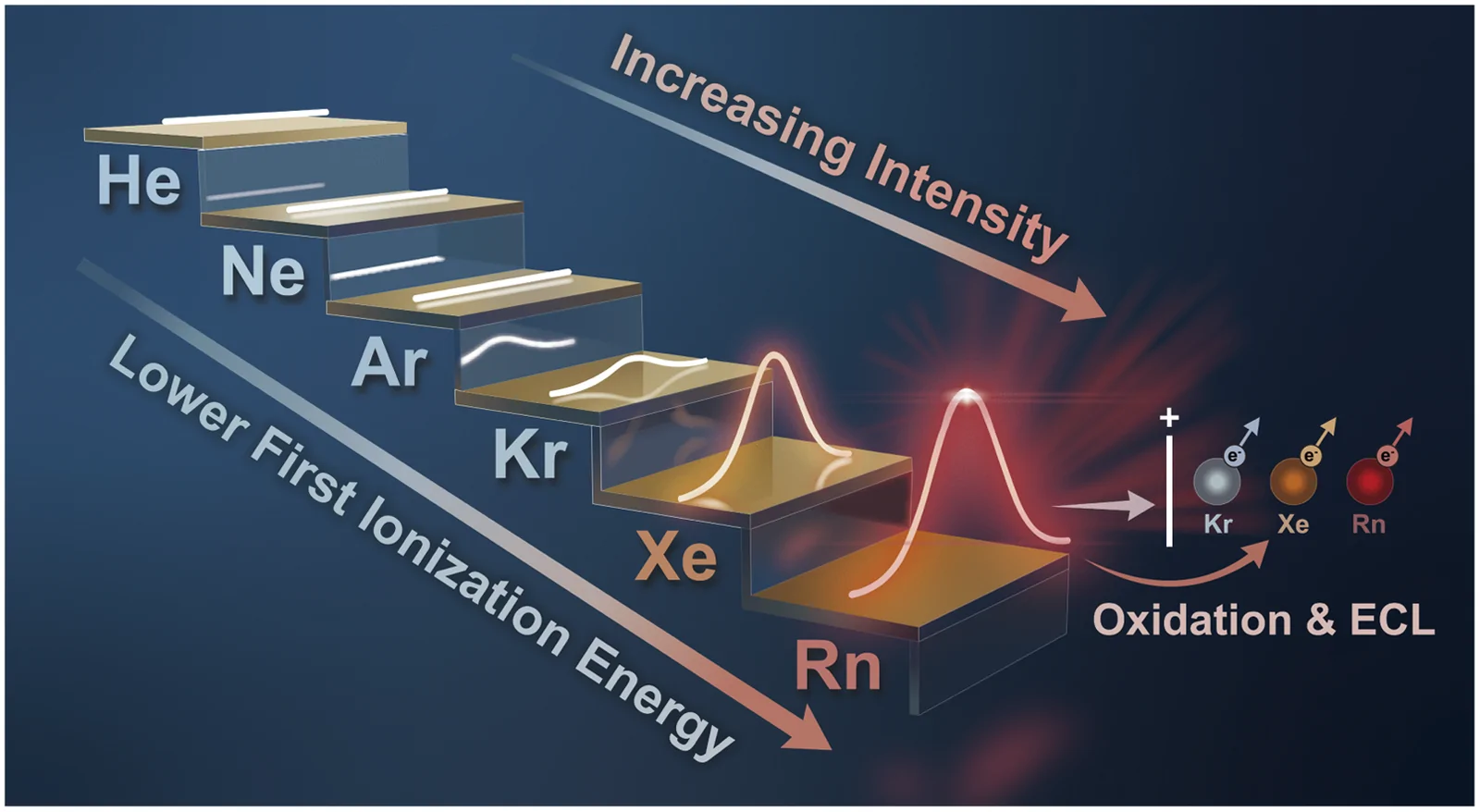

## Introduction

Noble gases are widely applied in various scenarios closely related to daily life, such as environmental analysis, laser technology, medical diagnosis, geological tracing, and commercial illumination.^1–4^ Among them, radon (Rn) is a radioactive noble gas and a widely distributed contaminant; it is the primary source of natural radiation exposure in the environment.^4^ Radon is considered the second leading cause of lung cancer after smoking, contributing to approximately 220 000 deaths annually worldwide. Radon is the decay product of ^232^Th, ^235^U and ^238^U in soil, rock, as well as building materials. It can enter buildings through some joints and cracks, where it can accumulate in indoor air.^5^ Anomalous variations in trace radon concentrations are potential precursors to seismic events, such as earthquakes and tsunamis.^6^ Regions exhibiting anomalously high radon concentrations are often associated with subsurface resources, including oil, mineral deposits, and groundwater.^7,8^ Additionally, the radioactive elements xenon (^127^Xe) and krypton (^85^Kr), which are produced by nuclear fission, are also known as key hazardous contaminants in nuclear fuel reprocessing, and their accurate determination is needed.^1^ Therefore, studies on noble gases exhibit obvious significance for applications in various fields including the environment, geoscience and others.

The noble gases are traditionally considered to be a series of elements with relatively low chemical reactivity, suggesting that they are unlikely to participate in chemical reactions. Electrochemical processes are significant chemical phenomena that can often produce excited states and generate electrochemiluminescence (ECL) signals at the surface of working electrodes.^9^ ECL is generated *via* electrochemical redox reactions that produce electronically excited luminophores and subsequent photon emission,^10,11^ and has been widely applied in clinical immunoassays,^12^ nucleic-acid sensing,^13^ environmental pollutant monitoring,^14^ and cellular bioimaging.^15^ In this study, we report the electrochemical redox and ECL mechanisms of noble gases (radon, xenon and krypton) for the first time. Among them, radon gives obvious ECL signals both in an annihilation state and in the presence of a co-reactant reagent, and exhibits an obvious free radical-solvent interaction-dependent ECL (FSDE) mechanism in an annihilation state. In comparison, xenon, which has redox properties similar to those of radon,^16,17^ cannot exhibit an ECL signal in an annihilation state due to its higher first ionization energy. Krypton, xenon and radon can all exhibit anodic ECL signals when a co-reactant tripropylamine (TPrA) is added. The key intermediates Rn/Xe˙^+^ and Rn/Xe˙^−^ are confirmed using high-performance liquid chromatography-mass spectrometry (HPLC-MS) and electron paramagnetic resonance (EPR) analyses, which provide evidence for their electrochemical redox and ECL processes. The ECL behaviors of these noble gases were employed for the monitoring of radon/xenon, and an interpretable machine learning strategy has been applied to decode the specific ECL signals of Rn and Xe for their sensitive and selective determination. Their practical detection in the environment is further confirmed in various kinds of atmospheres, such as natural caves, the indoor environment of buildings, basements, tunnels, and other environments, demonstrating the significance of this method in the fields of the environment, public health and geoscience.

## Results and discussion

### Electrochemical properties of radon

To characterize the electrochemical properties of noble gases, a suitable working electrode with good conductivity and a porous structure for gas adsorption must be chosen. Therefore, a carbon paper-supported electrode (Materials and Apparatus, SI) was employed as the working electrode in the three-electrode system. Its SEM image is presented in Fig. S1A, demonstrating its fibrous structure. To accommodate this electrode configuration, the ECL signals were obtained using a self-developed up-sensing ECL analysis system, as shown in Fig. S1B–D. The BET surface area value of this electrode was measured to be 11.4 m^2^ g^−1^, suggesting the presence of porous structures for adsorbing radon. The elemental analysis results (C: 83%; H: 0.15%; O: 0%; N: 0.08% and S: 0%) show that this electrode is mainly composed of carbon without functional groups, indicating that the adsorption of radon is a physical process. Obviously enhanced count rate values can be observed at 295, 352 and 609 keV in the γ energy spectra of the carbon paper-supported electrodes (Fig. 1A), indicating the adsorption of radon on the electrode.^18,19^

**Fig. 1:**
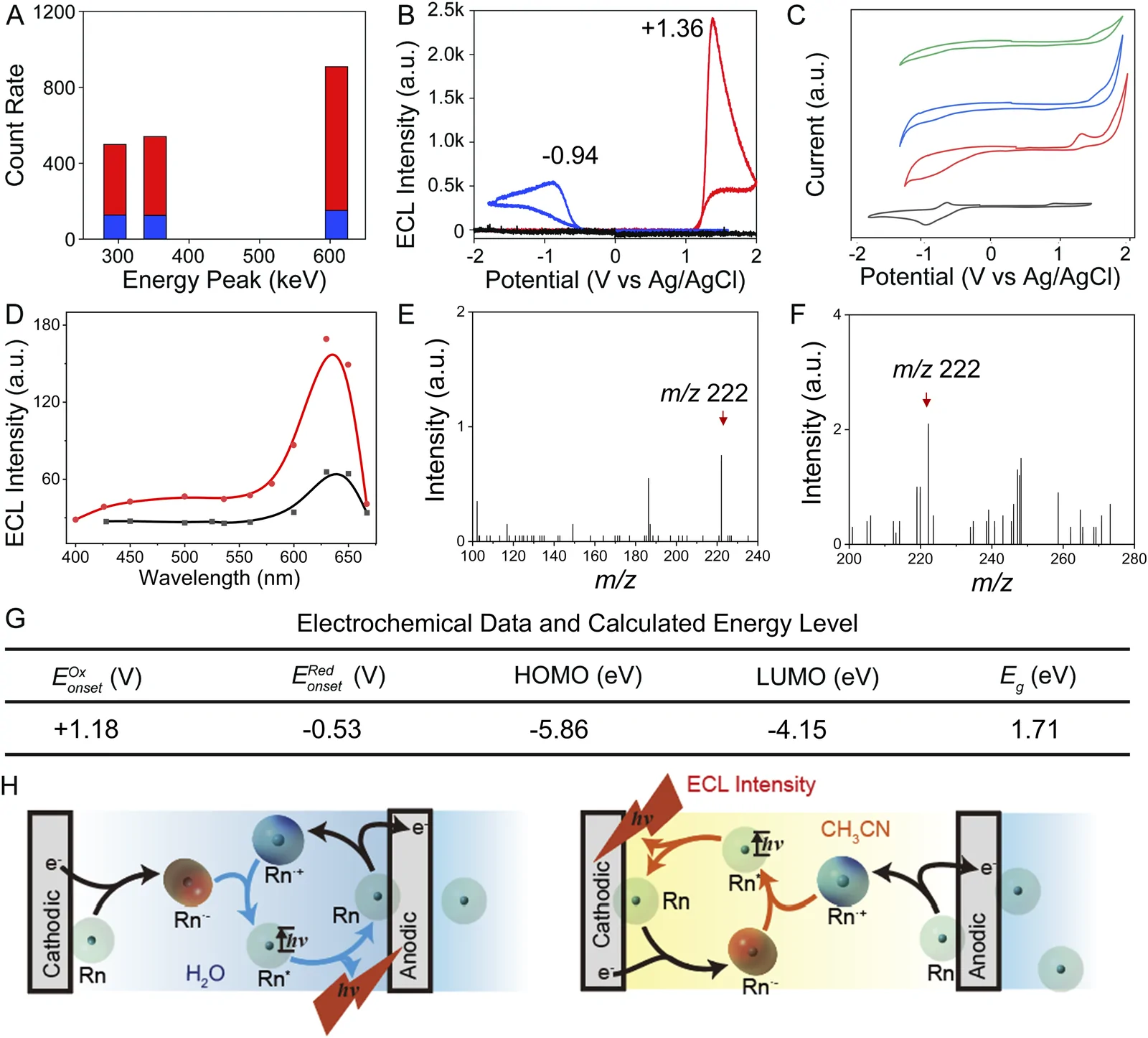
Electrochemical and ECL mechanism in annihilation state. (A) Count rate values at 295, 352 and 609 keV of the γ energy spectra of carbon paper-supported electrodes: blank (blue) and radon (2000 Bq m^−3^)-treated (red); (B) ECL signals of xenon (black), 2000 Bq m^−3^ radon-treated carbon paper-supported electrode in 0.1 M PBS (pH = 7.4) (red) and 2000 Bq m^−3^ radon-treated carbon-paper-supported electrode in CH_3_CN with 0.1 M Bu_4_NBF_4_ (blue); (C) cyclic voltammograms of carbon paper-supported electrodes: blank electrode (green), electrode with Pb powder (blue), Rn (2000 Bq m^−3^)-treated electrode in PBS (red), and Rn (2000 Bq m^−3^)-treated electrode in CH_3_CN (black); (D) 0.1 M PBS solution (pH = 7.4) (red) and CH_3_CN with 0.1 M Bu_4_NBF_4_ (black). Scan rate: 100 mV s^−1^, PMT: 900 V; (E) positive-ion HPLC-MS spectrum of the anodic Rn/methanol sample; (F) negative-ion HPLC-MS spectrum of the cathodic Rn/acetonitrile-BF_3_ sample; (G) electrochemical data and calculated energy level of radon; (H) mechanism of the free radical-solvent interaction-dependent ECL of radon on the three-electrode system.

The carbon paper-supported electrodes with adsorbed radon show ECL signals in an annihilation state in phosphate-buffered saline (PBS) solution and CH_3_CN (Fig. 1B, red and blue), while the blank electrode does not give an ECL signal in either of the solvents (Fig. S2A and B). These results confirm that the ECL signals in Fig. 1B can be assigned to radon. Radon shows an obvious anodic ECL signal in aqueous solvent and gives an emission peak at around +1.36 V. On the contrary, it gives a cathodic ECL signal in the solvent CH_3_CN at around −0.94 V (Fig. 1B). In the cyclic voltammetry (CV) data, considering the relatively long half-life of ^210^Pb (22.3 years), a long-lived progeny of radon, an electrode containing Pb powder was examined as a control (Fig. 1C, blue). No corresponding redox peaks were observed at the potentials of the Rn-associated responses, excluding ^210^Pb as their origin. In contrast, the Rn-treated electrode exhibited an oxidation peak at +1.32 V in PBS (Fig. 1C, red), and a reduction peak at −0.92 V in CH_3_CN (Fig. 1C, black). These results further confirm that the ECL signals in Fig. 1B can be assigned to the electrochemical redox reaction of radon. To further investigate the electrochemical processes, scan-rate-dependent CV measurements were performed over 0.02–0.50 V s^−1^ (Fig. S3). Because the Rn-associated anodic and cathodic responses were preferentially observed in PBS and CH_3_CN, respectively, the anodic process was investigated in PBS, whereas the cathodic process was investigated separately in CH_3_CN. The anodic peak current increased linearly with *v*^1/2^, giving a slope of approximately 0.046 A (V s^−1^)^−1/2^ and *R*^2^ = 0.99959 (Fig. S3A and B). Similarly, the magnitude of the cathodic peak current showed a linear dependence on *v*^1/2^, with a slope of approximately 0.043 A (V s^−1^)^−1/2^ and *R*^2^ = 0.99967 (Fig. S3C and D). These relationships indicate a substantial mass-transport-coupled contribution to both responses.^20–22^

The modest difference between the anodic and cathodic slopes can be attributed to their different reaction media and radical-ion stabilization. DFT calculations showed that Rn˙^+^ is more strongly stabilized than Rn˙^−^ in water (−28.54 *versus* −10.18 kcal mol^−1^), whereas Rn˙^−^ is slightly more stable than Rn˙^+^ in CH_3_CN (−43.98 *versus* −39.78 kcal mol^−1^, Fig. 2 and S7). These results provide a qualitative explanation for the preferential anodic response in PBS and cathodic response in CH_3_CN.^23^ EPR results further confirmed the generation of Rn˙^+^/Rn˙^−^ (Fig. S4). The small slope difference may reflect the combined effects of radical formation and stabilization, solvent-dependent diffusion, gas transport, ionic conductivity and interfacial structure.^24^ Accordingly, the two features are described as separate Rn-associated anodic and cathodic electron-transfer processes rather than the paired peaks of a conventional reversible redox couple. The ECL spectra of radon were also obtained, as shown in Fig. 1D. Radon gives similar ECL emission peaks around 640 nm in aqueous (red line) and CH_3_CN (black line) solutions, demonstrating the same excitation state in these two ECL processes. A reasonable ECL mechanism in an annihilation state can be summarized as follows:

1 Rn − e^−^ → Rn˙^+^

2 Rn + e^−^ → Rn˙^−^

3 Rn˙^+^ + Rn˙^−^ → Rn* + Rn

4 Rn* → Rn + *hv*

**Fig. 2:**
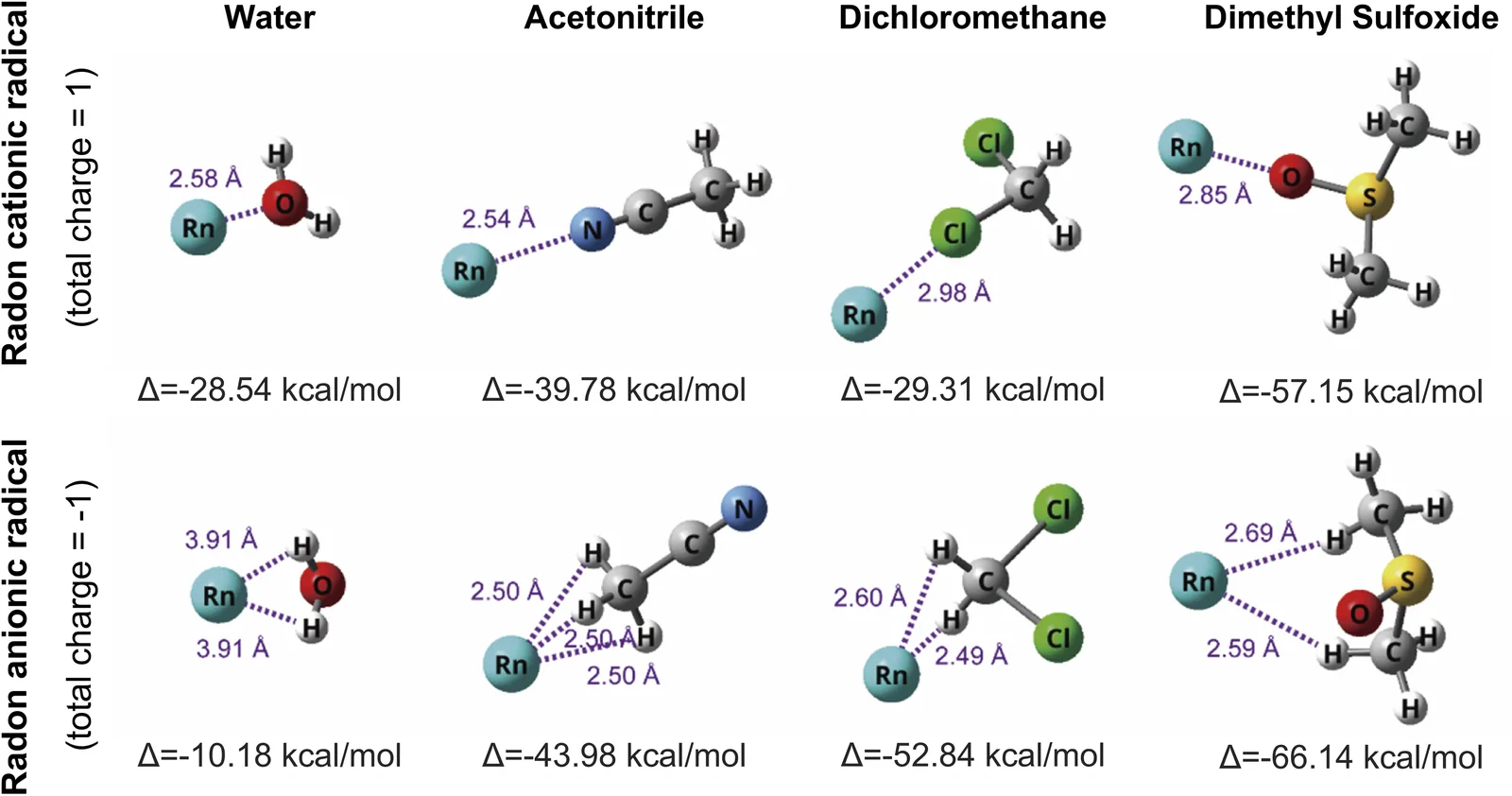
Binding energy between Rn˙^−^/Rn˙^+^ and four solvent molecules obtained *via* DFT calculations.

To confirm the key intermediates Rn˙^+^/Rn˙^−^ in eqn (1) and (2), HPLC-MS measurements were performed with CH_3_OH and BF_3_ as stabilizers for the free radicals, respectively. Density functional theory (DFT) calculations show that CH_3_OH and BF_3_ can combine well with Rn˙^+^/Rn˙^−^*via* weak interactions (such as the dispersion and Coulomb forces), respectively, providing strong stabilization of these free radicals (Fig. S5).^25–27^ Compared to the blank samples, the Rn-exposed samples exhibit new peaks in the HPLC data, indicating the generation of new substances (red squares in Fig. S6).^28^ Positive- and negative-ion MS subsequently displayed nominal-mass signals at *m*/*z* = 222 (Fig. 1E and F), which demonstrate the existence of Rn˙^+^/Rn˙^−^ in the electrochemical redox processes, respectively.

Based on the CV data in Fig. 1C, the electrochemical data for radon were calculated according to the method reported previously in the literature (Fig. 1G).^29,30^ The HOMO and LUMO energy levels of radon were determined to be −5.86 eV and −4.15 eV, respectively, yielding an electrochemical band gap of 1.71 eV. Based on the ECL spectra in Fig. 1D, the optical band gap value was calculated to be 1.94 eV using the formula *E*_g_ = 1239.8/*λ* (eV) (*λ* = 640 nm), which is similar to the electrochemical band gap. This result demonstrates the reliability of the electrochemical data of radon.

### Free radical-solvent interaction-dependent ECL (FSDE) mechanism

It can be observed that radon exhibits different ECL behavior in H_2_O and CH_3_CN, respectively (Fig. 1B and H). To explain this phenomenon, the interactions between Rn˙^+^/Rn˙^−^ free radicals and solvent molecules have been characterized *via* DFT calculations (Fig. 2 and S7). The results suggest that both Rn˙^−^ and Rn˙^+^ can combine with the solvent molecules H_2_O and CH_3_CN. Among them, the interaction between H_2_O and Rn˙^+^ is more stable than that between H_2_O and Rn˙^−^, which may make the electron-accepting process of Rn˙^+^ more difficult (eqn (3)) in the cathodic range and be beneficial for anodic ECL emission in PBS solution. On the contrary, the interaction between CH_3_CN and Rn˙^−^ is more stable than that between CH_3_CN and Rn˙^+^ (Fig. 2). This interaction may make the electron transfer from Rn˙^−^ to Rn˙^+^ more difficult in anodic scanning, which induces the cathodic ECL in CH_3_CN. The interaction of Rn˙^−^/Rn˙^+^ with CH_3_CN is stronger than that with H_2_O (Fig. 2), which may reduce the dissociation of Rn˙^−^/Rn˙^+^ on the electrode surface and thereby inhibit the annihilation reaction between Rn˙^−^ and Rn˙^+^ (eqn (3)). As a result, a weaker ECL signal for radon is obtained in CH_3_CN than in H_2_O (Fig. 1B). Therefore, these results suggest that the interaction between radon free radicals and solvent molecules may be a key factor affecting the ECL of radon.

Two other organic solvents, CH_2_Cl_2_ (DCM) and DMSO, were selected to confirm the solvent-dependent ECL mechanism. As shown in Fig. 2, the interactions of Rn˙^−^ with DCM/DMSO are both more stable than those of Rn˙^+^. Similar to the ECL signals of radon in CH_3_CN, the signals in these solvents both appear in the cathodic range (Fig. S8). Additionally, the ECL intensities in DCM/DMSO are lower than that in CH_3_CN. This phenomenon can be attributed to the stronger interactions between DCM/DMSO and Rn˙^−^ (Fig. 2), which block the annihilation ECL process. Thus, these results confirm the FSDE mechanism of radon.

Chronoamperometry (CA) was further employed to confirm the ECL mechanism of radon. As shown in Fig. 3A, radon does not exhibit an ECL signal in PBS solution under the initial +2 V potential even though Rn˙^+^ is produced, which can be attributed to the lack of Rn˙^−^ in this process. When the potential is switched to −2 V, an ECL signal is observed due to the formation of Rn˙^−^, which undergoes an annihilation reaction with Rn˙^+^ to generate the excited state Rn* through eqn (3). When the initial potential is changed to −2 V, a strong ECL signal appears in the range of +2 V (Fig. 3B). This phenomenon indicates a process similar to that shown in Fig. 3A.

**Fig. 3:**
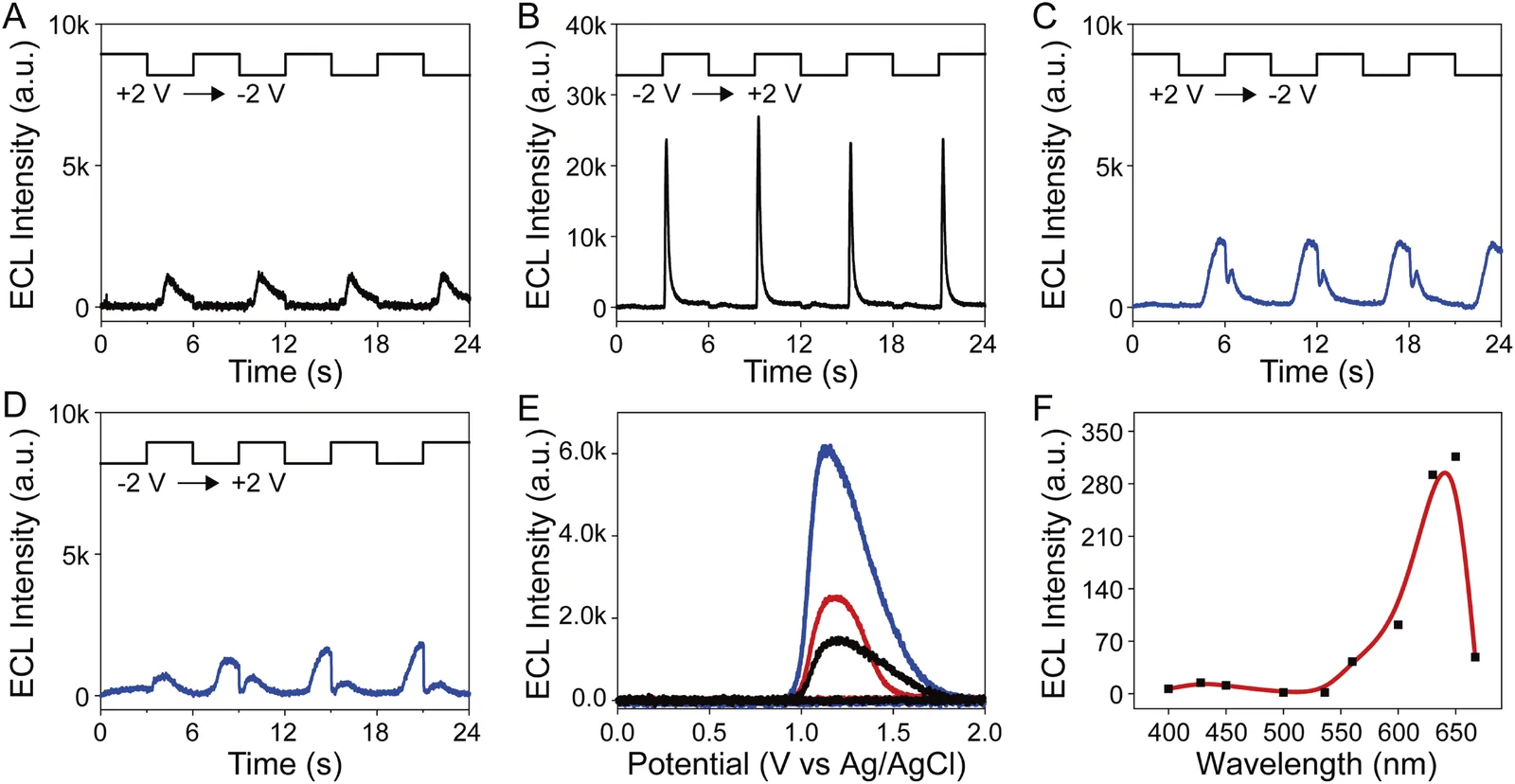
CA data and ECL of radon with a co-reactant reagent. ECL signals of the blank electrode using the CA method in 0.1 M PBS solution (black) and CH_3_CN with 0.1 M Bu_4_NBF_4_ (blue) as a function of the scan potential with potentials of (A and C) +2 V and −2 V, (B and D) −2 V and +2 V, with 3 s per potential. PMT: 900 V; (E) ECL signal of 2000 Bq m^−3^ radon-treated carbon paper-supported electrodes allowed to decay for different times: 0 d (blue), 3 d (red), and 5 d (black); (F) ECL spectrum of a 2000 Bq m^−3^ radon-treated carbon paper-supported electrode. Electrolyte: 0.1 M pH 7.4 PBS with 25 mM TPrA, scan rate: 100 mV s^−1^, PMT: 600 V.

Compared to its PBS solution, radon exhibits obviously different ECL behavior in CH_3_CN (Fig. 3C and D). Radon gives ECL peaks in both the −2 V and +2 V ranges, which indicates that Rn˙^−^/Rn˙^+^ may not completely annihilate in each scan period (3 s) in CH_3_CN. This phenomenon may be attributed to the higher binding energy of Rn˙^−^/Rn˙^+^ with CH_3_CN than with H_2_O. The ECL peaks in PBS solution are narrow and sharp compared with those in CH_3_CN, suggesting a faster ECL process in PBS than in CH_3_CN. This result can also confirm the stronger combination of Rn˙^−^/Rn˙^+^ with CH_3_CN than with H_2_O (Fig. 2). As shown in Fig. S9, blank electrodes do not give ECL signals in either PBS or CH_3_CN solution, indicating that the ECL signals in Fig. 3A–D can be assigned to radon. Furthermore, radon gives a higher ECL signal in the anodic range in PBS solution, while its cathodic ECL signal is higher in CH_3_CN solution, which is similar to Fig. 1B. These results further confirm the ECL mechanism involving an annihilation state (eqn (1)–(4)).

### Co-reactant ECL of radon, xenon and krypton

An enhanced anodic ECL signal is achieved with the addition of tripropylamine (TPrA) as a co-reactant reagent (blue, Fig. 3E) to give an emission peak at around +1.36 V with an onset potential of +1.11 V, which is similar to those of the annihilation state (Fig. 1B, red). TPrA exhibits an oxidation peak at around +1.29 V with an onset potential of +0.92 V (Fig. S10A); these values are lower than those of radon (Fig. 1C). This result indicates that the ECL process of the radon/TPrA system begins with the oxidation of TPrA. Compared with those of the annihilation state, the ECL spectrum of the radon/TPrA system gives a similar emission peak at around 640 nm (Fig. 3F). This result suggests the same excited state in these two ECL processes. Therefore, a reasonable anodic ECL process for radon can be summarized as below:

5 TPrA − e^−^ → TPrAH˙^+^

6 TPrAH˙^+^ − H^+^ → TPrA˙

7 Rn − e^−^ → Rn˙^+^

8 Rn˙^+^ + TPrA˙ → Rn* + Pr_2_N^+^=CHCH_2_CH_3_

9 Rn* → Rn + *hv*

The ECL signals of carbon paper-supported electrodes with adsorbed radon were measured after different decay times (Fig. 3E). The ECL intensity of radon gradually decreases with the increase in decay time from 0 to 5 days. This result confirms that the ECL signal can be assigned to the electrochemical redox reaction of radon. To exclude the possibility of potential luminescent species induced by radiation, such as ROS species, tritium water was added to the ECL system to obtain a much higher radiation activity than that of radon (144 kBq L^−1^, Fig. S10B). In contrast to the carbon paper-supported electrode treated with 2000 Bq m^−3^ radon, no obvious ECL signal can be observed without radon treatment, even with the addition of tritium water. This phenomenon indicates that the ECL signal can be assigned to the electrochemical redox reaction of radon.

Additionally, xenon and krypton were observed to give ECL signals with TPrA as a co-reactant reagent among the other five noble gases (Fig. 4A), although they cannot give ECL signals in an annihilation state. Xenon gives an ECL signal at around +1.40 V (Fig. 4A, red), which is similar to its oxidation peak (+1.40 V) in the CV data (Fig. 4B, blue). The emission peak of xenon appears at around 600 nm (Fig. 4C). Krypton also gives a weak ECL signal at around +1.48 V (Fig. 4A, blue), which is similar to its oxidation peak (+1.47 V, Fig. 4B, red). He/Ne/Ar do not exhibit ECL signals (Fig. 4A) due to their lack of electrochemical redox properties (Fig. 4B, black, purple and green). The oxidation potentials of Kr, Xe and Rn follow an obvious trend of Kr > Xe > Rn. This result can be attributed to the gradually reduced first ionization energy value from Kr to Rn (Table S1), which indicates that the oxidation capacity of these three noble gases is Rn > Xe > Kr. This can also explain the weak ECL signal of Kr compared with those of Xe and Rn. The oxidation potential of TPrA (Fig. S10A, +1.29 V), which is lower than that of Xe, shows that the ECL process of Xe begins with the oxidation of TPrA. Therefore, Xe shares the same ECL mechanism as Rn, as shown below:

10 TPrA − e^−^ → TPrAH˙^+^

11 TPrAH˙^+^ − H^+^ → TPrA˙

12 Xe/Rn − e^−^ → Xe/Rn˙^+^

13 Xe/Rn˙^+^ + TPrA˙ → Xe/Rn* + Pr_2_N^+^=CHCH_2_CH_3_

14 Xe/Rn* → Xe/Rn + *hv*

**Fig. 4:**
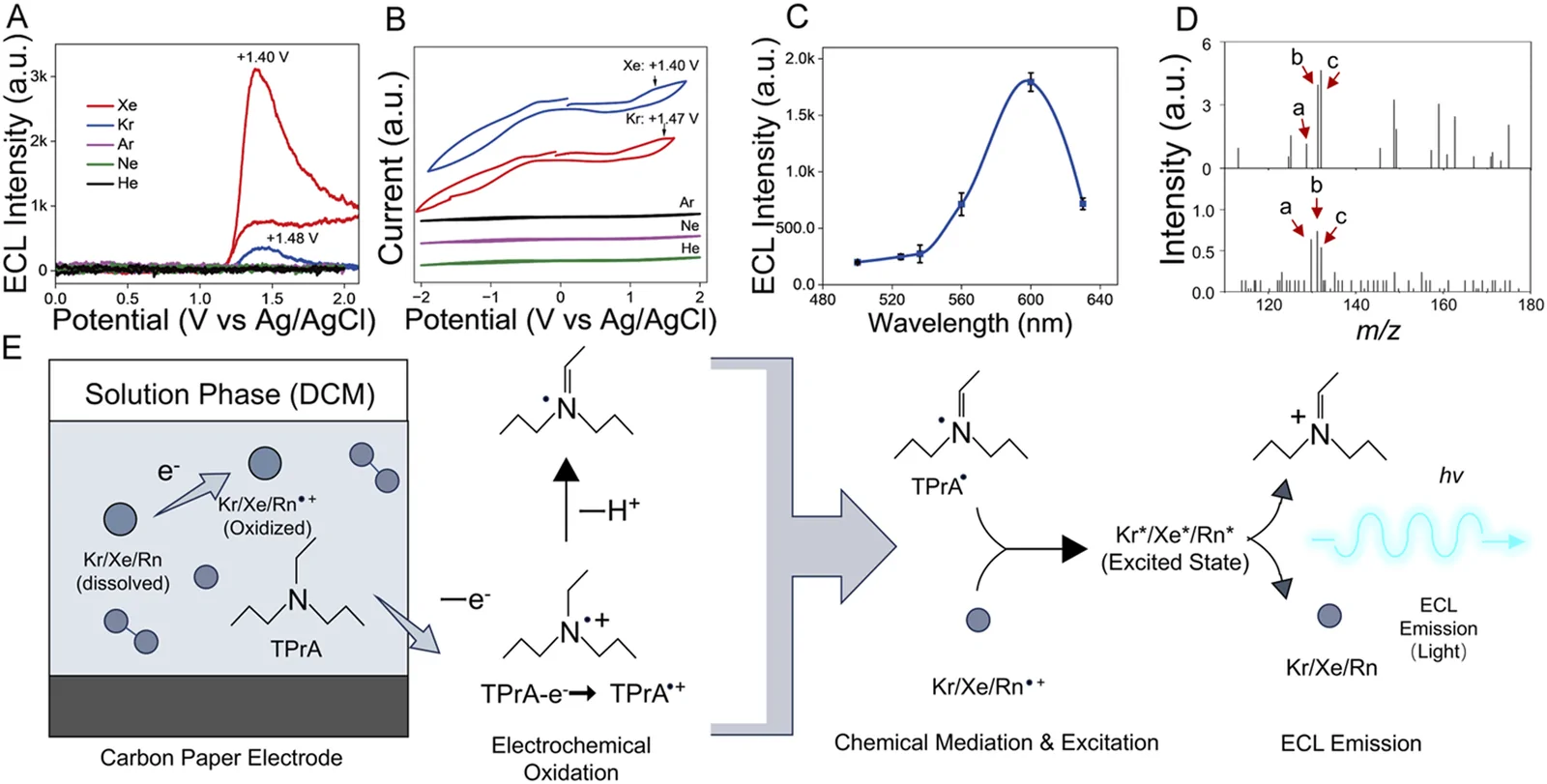
ECL of xenon and krypton. (A) ECL signals of He, Ne, Ar, Kr and Xe with 25 mM TPrA and (B) CV data of He, Ne, Ar, Kr and Xe in CH_2_Cl_2_ with 0.1 M Bu_4_NBF_4_; (C) ECL spectrum of Xe; (D) HPLC-MS data of a methanolic solution obtained from the paper-supported electrode with adsorbed Xe after CV scanning. Upper: negative-ion MS of Xe; lower: positive-ion MS of Xe. (a–c) correspond to *m*/*z* = 129, 131, and 132, respectively. (E) General schematic mechanism of TPrA-mediated ECL of noble gases (Kr/Xe/Rn) on a carbon paper electrode in CH_2_Cl_2_.

Similar to radon, the key intermediate free radicals of xenon (Xe˙^+^/Xe˙^−^) were also confirmed using HPLC-MS. DFT calculations indicate that Xe˙^+^/Xe˙^−^ can be stabilized by methanol and BF_3_, respectively (Fig. S5). Both Xe˙^+^ and Xe˙^−^ were also confirmed from the MS data (*m*/*z* = 129, 131, and 132) of the three main natural Xe isotopes (^129^Xe, ^131^Xe and ^132^Xe), respectively (Fig. 4D and S11).^31^ This result confirms the reliability of the ECL process of Xe. Meanwhile, Kr gives a weak ECL signal, indicating the difficulty of oxidizing Kr. This may be the reason why we cannot observe the Kr˙^+^ intermediate state in this work. Considering its higher oxidation potential compared to TPrA, we suppose that Kr probably shares the same co-reactant ECL process as Xe and Rn (Fig. 4E).

### Application in environmental monitoring

The ECL emission property of radon can be employed for the accurate determination of trace radon and xenon in the environment. The WHO standard (WHO Handbook on Indoor Radon: A Public Health Perspective) as well as the standards of some national governments (such as the National Standard of P. R. China: GB/T 16146-2015) mandate an extreme limit on the radon concentration in an indoor environment of 100 Bq m^−3^ (17 pg L^−1^), indicating the high accuracy requirement for radon detection. To date, known radon determination technologies mostly involve radiation detection and chemical sensors. Radiation detection techniques focus on determining the α or γ-spectrometry of radon and its decay products (^214^Po, ^218^Po and so on),^32,33^ which are the regular determination technologies in the WHO standard (WHO Handbook on Indoor Radon: A Public Health Perspective). Chemical sensors are mainly focused on the determination of ^210^Pb, a relatively stable decay product of radon with a half-life of 22.3 years.^34–36^ In contrast to these methods, the ECL signal is caused by the electrochemical redox reaction of noble gases. This guarantees high selectivity, which can be regarded as the most important potential advantage of this method. Additionally, the blank carbon paper-supported electrode exhibits no obvious ECL signal (black line, Fig. 5A), suggesting that there is almost no background in radon determination.

**Fig. 5:**
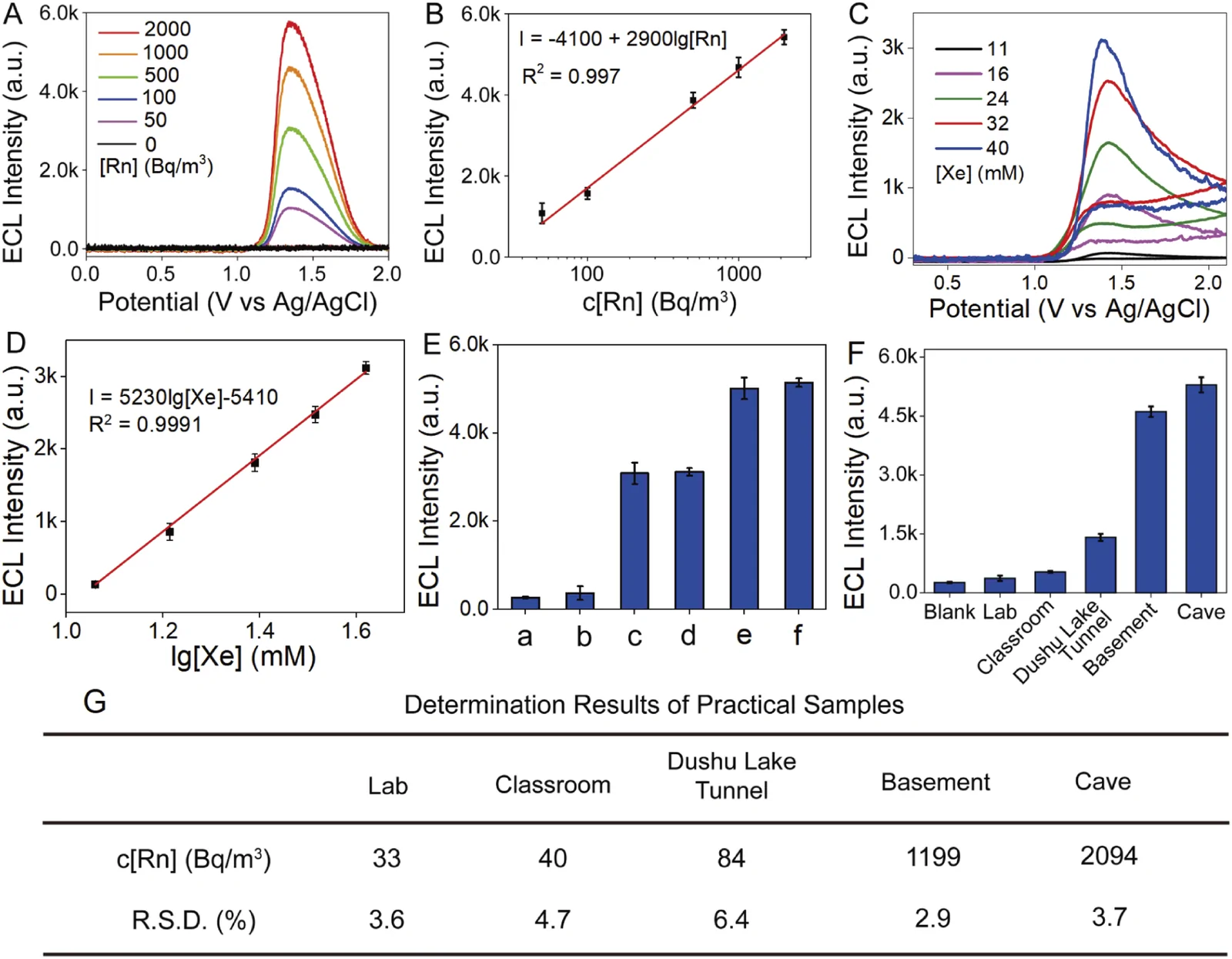
The application in radon determination. ECL signals of carbon paper-supported electrodes treated by radon (A) and xenon (C) with different concentrations; calibration curve of ECL intensity *versus* lg[Rn] (B) and lg[Xe] (D) values; (E) ECL intensity of carbon paper-supported electrodes treated by (a) blank, (b) interferences (CH_2_Cl_2_, toluene, tetrahydrofuran, EtOH, ethyl ether, cyclohexane, NH_3_, H_2_O with saturated vapor pressure), (c) interference with 40 mM Xe, (d) 40 mM Xe, (e) 1000 Bq m^−3^ radon with interferences and (f) 1000 Bq m^−3^ radon; (F) ECL intensity of carbon paper-supported electrodes treated by practical atmosphere samples and (G) the measurement results. Electrolyte: 0.1 M pH 7.4 PBS with 25 mM TPrA, scan rate: 100 mV s^−1^, PMT: 600 V, adsorption time: 8 h.

The best adsorption time before ECL measurement was studied, as shown in Fig. S12; 8 h was found to be the best adsorption time for radon determination. After adsorption under different radon concentrations, the carbon paper-supported electrode exhibits gradually increasing ECL signals (Fig. 5A and B) to give an LOD value of 8 mBq m^−3^ (64 mBq h m^−3^) as determined using the formula given in the SI. The ultra-low LOD value demonstrates the high sensitivity of this radon detection method (Table S2), which can be attributed to the extremely low background of the blank carbon paper-supported electrode. The ECL behavior of xenon can also be applied to the detection of xenon, as shown in Fig. 5C and D. To demonstrate the selectivity of radon/xenon detection, some typical gaseous interferents were chosen and investigated at saturated vapor pressure, including CH_2_Cl_2_ (a halohydrocarbon), toluene (an aromatic hydrocarbon), tetrahydrofuran (a cyclic ether), EtOH (an alcohol), ethyl ether (an ether), cyclohexane (an aliphatic hydrocarbon), NH_3_ and H_2_O (humidity). As shown in Fig. 5E, these interferents do not affect the radon and xenon detection results, confirming the good selectivity.

Considering the wide distribution of radon in the environment, this detection method was further applied to trace radon determination in practical atmospheric samples. The practical samples were collected from a common classroom, a basement without good ventilation and a common chemical lab in Soochow University, from the Dushu Lake Tunnel of Suzhou Industrial Park, and from a natural cave in the Linwudong Park of Suzhou (Fig. 5F and G), respectively. It can be observed that the basement sample showed a high radon concentration of 1199 Bq m^−3^ (Fig. 5G), almost 12 times higher than the WHO standard (100 Bq m^−3^). The result obtained using a commercially available fast radon monitor was similar at 1285 Bq m^−3^ (Fig. S13), indicating the reliability of the ECL detection method. The radon concentration of the Dushu Lake Tunnel underground structure (84 Bq m^−3^) was sharply lower than that of the basement (1199 Bq m^−3^), and this lower concentration can be attributed to the good ventilation in this tunnel. The radon concentration in the classroom was measured to be 40 Bq m^−3^, which falls within the normal range of radon concentrations in Suzhou (40–60 Bq m^−3^) and could not be determined using the fast radon monitor. The radon concentration in the chemical lab (33 Bq m^−3^) was lower than that of the classroom, which can be attributed to the ventilation function of the fume hoods. In contrast, the natural cave in Linwudong Park gave the highest radon concentration of 2094 Bq m^−3^, which may be attributed to natural radionuclides such as uranium-238 and radium-226 in the rocks of the cave. They can continuously release radon through their decay process and accumulate in the cave without good ventilation. These results indicate that good ventilation is important for reducing radon accumulation in indoor environments.

### Machine-learning-based analysis

To achieve rapid classification and quantitative analysis of Rn and Xe based on their ECL behaviors, a machine learning (ML) approach was employed.^37,38^ Given the safety constraints of manipulating radioactive radon, the acquired ECL dataset is inherently limited (*n* = 81) (Fig. 6A). First, six key physical features, including maximum intensity, peak position, full width at half maximum (FWHM), peak bottom width, integrated peak area, and skewness, were extracted from the ECL waveforms (Fig. 6A). A Pearson correlation heatmap was then constructed to evaluate the multicollinearity among these parameters (Fig. 6B). Unsupervised *t*-SNE visualization revealed distinct clustering of the Rn and Xe populations, indicating the intrinsic separability of the high-dimensional features (Fig. 6C).^39^ To mitigate overfitting under small-sample conditions, three regularized ML algorithms, logistic regression (LR), support vector machine (SVM), and random forest (RF), were trained utilizing a stratified 5-fold cross-validation pipeline.^40^ All three models demonstrated exceptional diagnostic performance on the test cohorts, achieving 100% classification accuracy without false assignments (Fig. 6D and S14), supported by area under the curve (AUC) values exceeding 0.99 (Fig. S15).

**Fig. 6:**
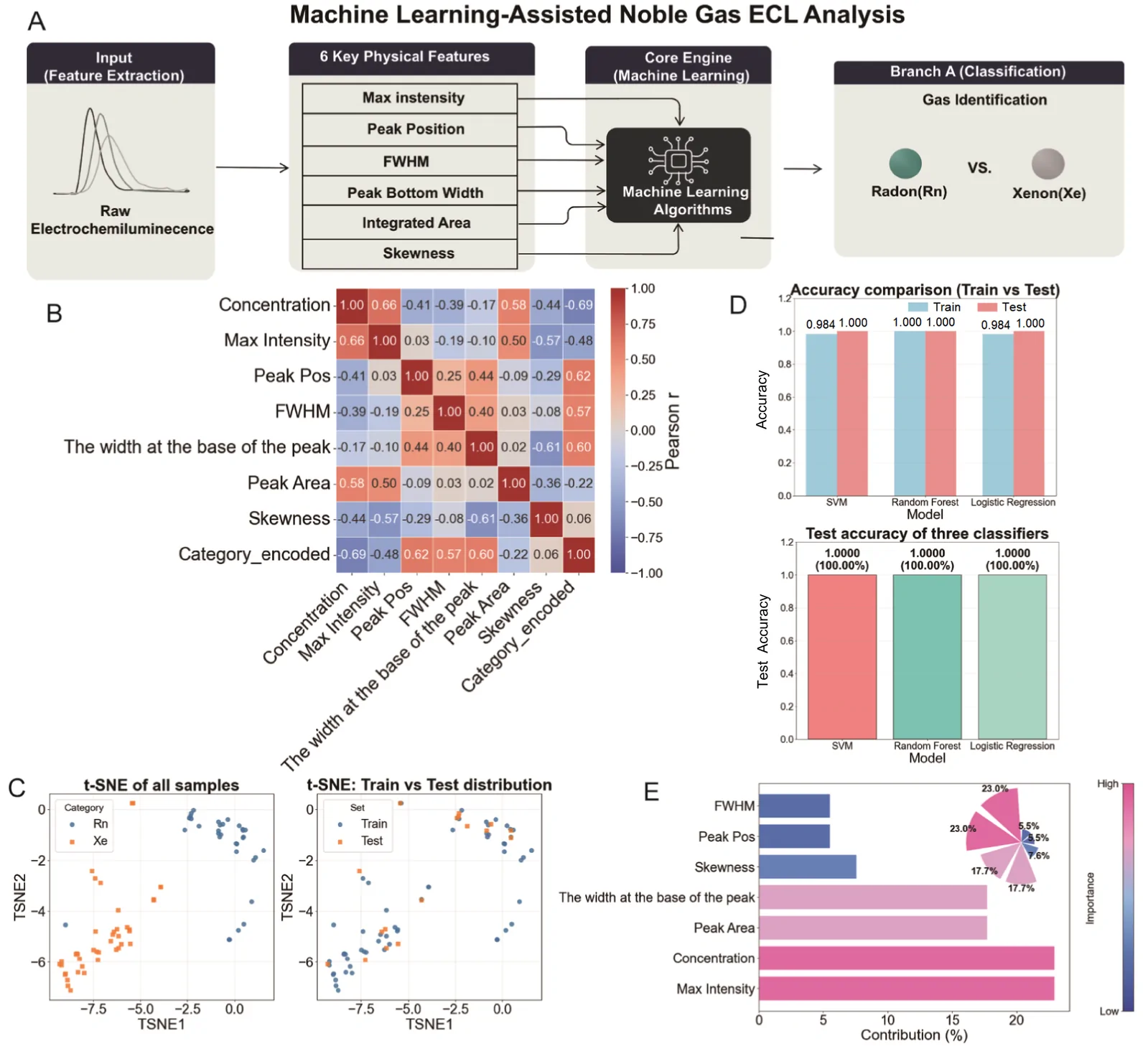
Machine-learning-assisted analysis of noble gas (Rn and Xe) electrochemiluminescence. (A) Schematic illustration of the machine learning workflow; (B) Pearson correlation heatmap illustrating the interdependencies among the six extracted physical features; (C) *t*-SNE visualization demonstrating the inherent separability of Rn and Xe in the small-sample dataset; (D) performance evaluation metrics of the LR, SVM, and RF classifiers; (E) SHAP rose plot identifying maximum intensity and integrated area as the dominant features driving the classification.

Finally, to circumvent the “black-box” nature of the algorithms and extract physically meaningful insights,^41^ SHapley Additive exPlanations (SHAP) analysis was applied to the RF model (Fig. 6E).^42^ SHAP interpretation identified maximum intensity and integrated peak area as the dominant features driving the classification.^43^ This data-driven result corresponds with the distinct electrochemical thermodynamics of Rn and Xe, offering an interpretable bridge between phenomenological ECL signals and noble gas properties.

## Conclusion

This work reports the electrochemical redox and ECL mechanism of noble gases for the first time. Among six noble gases, Rn/Xe/Kr exhibit electrochemical redox reactions to drive ECL emission, and the key intermediates Rn˙^+^/Xe˙^+^ were identified using HPLC-MS to confirm their electroredox and ECL processes. Only radon exhibits an ECL signal in an annihilation state with an obvious FSDE mechanism in different solvents, which has been studied by DFT calculations. The ECL behavior corresponds to the chemical properties of radon and xenon themselves, indicating the advantage of potentially high selectivity in radon determination applications. The ECL behavior of radon was successfully applied in accurate radon determination in various practical environmental samples, including samples from a natural cave, an underground structure, and a common indoor environment, with an ultra-low LOD of 8 mBq m^−3^ and high selectivity. An interpretable machine learning strategy was employed to decode the specific ECL signals of Rn and Xe to realize their rapid and accurate determinations. These results indicate the significance of this study in the fields of the environment, geoscience, and public health.

## Author contributions

Z. Wang conceived the study and wrote the manuscript. W. Zhao performed the experiments, carried out the machine-learning analysis and contributed to manuscript writing. Q. Sun performed the DFT calculations. Y. Li and C. Li performed the experiments. M. Xu contributed to data analysis and discussion. J.-J. Xu and D. Hua supervised the project and revised the manuscript. All authors discussed the results and approved the final manuscript.

## Conflicts of interest

There are no conflicts to declare.

## Supplementary Material

SC-OLF-D6SC04632K-s001

## Data Availability

The data supporting this article are included within the article and its supplementary information (SI) including further details on experimental procedures, computational details, electrochemical and ECL data, machine learning data and so on. The code used for the machine-learning analysis is publicly available on GitHub at https://github.com/imrealKen/Machine-Learning-Classification-for-Signal-Features. Supplementary information is available. See DOI: https://doi.org/10.1039/d6sc04632k.
