# Supplementary material for: Noble gas electrochemistry-driven luminescence integrated with machine learning for environmental monitoring
Source: Chem Sci. 2026 Oct 9. Online ahead of print. doi: 10.1039/d6sc04632k (PMC13650928; doi:10.1039/d6sc04632k)
Supplement: Supplementary file 1 [file SC-OLF-D6SC04632K-s001.pdf]

Supplementary Information (SI) for Chemical Science.
This journal is © The Royal Society of Chemistry

Electronic Supplementary Information

Noble Gas Electrochemistry-Driven Luminescence Integrated with Machine Learning for Environmental Monitoring

Ziyu Wang,‡ [a] Wenxuan Zhao,‡ [a] Qiao Sun,‡ [a] Yulin Li,[a] Chengqi Li,[a] Meiyun Xu,[a] Jing-Juan Xu,* [b] Daoben Hua*

[a]

[a] Dr. Z. Wang, W. Zhao, Prof. Q. Sun, Y. Li, C. Li, Dr. M. Xu and Prof. D. Hua
Jiangsu Key Laboratory of Radiation Injury Prevention and Hazardous Substance Control, School for Radiological and Interdisciplinary Sciences (RAD-X), Collaborative Innovation Center of Radiological Medicine of Jiangsu Higher Education Institutions, Soochow University, 199 Ren'ai Road, Suzhou, 215123, P. R. China.
E-mail: dbhua_lab@suda.edu.cn (D. Hua)

[b] Prof. J.-J. Xu
State Key Laboratory of Analytical Chemistry for Life Science, School of Chemistry and Chemical Engineering, Nanjing University, Nanjing 210023, P. R. China.
E-mail: xujj@nju.edu.cn (J.-J. Xu)

[‡] These authors contributed equally to this work.

[*] Corresponding Author.

Table of Contents

1. Experimental Procedures ………… S3

Materials and Apparatus ………… S3

Methods ………… S3

2. Supplementary Figures ………… S6

Fig. S1. Photographs of carbon paper-supported electrode and ECL device ………… S6

Fig. S2. ECL of carbon paper-supported electrode in PBS & CH_3CN about Rn ………… S7

Fig. S3. Scan-rate-dependent electrochemical characterization of Rn-treated carbon paper-supported electrodes ………… S8

Fig. S4. EPR spectra supporting electrochemically generated Rn-associated radical species ………… S9

Fig. S5. DFT-calculated stabilization of xenon and radon ions by CH_3OH and BF_3 ………… S10

Fig. S6. HPLC data of paper-supported electrode ………… S11

Fig. S7. Charge distribution *via* DFT calculations ………… S12

Fig. S8. ECL of carbon paper-supported electrode in organic solvents ………… S13

Fig. S9. ECL signals of blank electrode by CA method ………… S14

Fig. S10. Electrochemical analysis of aqueous solutions ………… S15

Fig. S11. HPLC data of paper-supported electrode ………… S16

Fig. S12. ECL intensity of carbon paper-supported electrode treated for 0 to 10 h ………… S17

Fig. S13. Detection result of radon concentration in the basement ………… S18

Fig. S14. Confusion matrices of the three machine learning classifiers ………… S19

Fig. S15. Receiver operating characteristic (ROC) curves of the machine learning framework ………… S20

3. Supplementary Tables ………… S21

4. Notes and references ………… S22

Experimental Procedures

Materials and Apparatus.

All solvents and reagents were commercially available and of analytical reagent grade. 0.1 M Phosphate buffered saline (PBS) was purchased from Jiangsu KeyGEN Bio TECH Co., Ltd. (Nanjing, China). Carbon paper-supported electrodes (type: HCP010N) was purchased from Suzhou Suke Lean Instrument Co., Ltd. (Suzhou, China). The carbon paper was produced using carbon fiber as the paper pulp. All other chemical reagents used (acetonitrile, dimethyl sulfoxide, dichloromethane and tripropylamine) and Bu_4NBF_4 were of analytical grade or better and were purchased from Sinopharm Chemical Reagent Co., Ltd. (Shanghai, China). Double-distilled water was used throughout the experiments.

Electrochemical and ECL spectra were conducted on an ECL analyzer equipped with a CHI-660E electrochemical station (supplied by the State Key Laboratory of Analytical Chemistry for Life Science, Nanjing University). ECL emission measurements were conducted on a self-developed ECL analyzer. The spectral width of the photomultiplier tube (PMT) was 350-650 nm. Radon concentration was measured by Radon monitor (NT8260, Shanghai Nuctest Instrument Co., Ltd., China). ^{222}Rn were collected from a radon chamber (HD-3) at Suzhou Medical College of Soochow University. Scanning electron microscope (SEM) was taken by Regulus 8230 (Hitachi, Ltd., Japan). The count rate was measured by High Purity Germanium (HPGe) gamma spectrometer (AMETEK Inc., the USA). BET was measured by QUADRASORB (Über Anton Paar, Austria). HPLC-MS analyses were performed using an Agilent 1260 HPLC system coupled to an Agilent 6120 mass spectrometer (Agilent Technologies, USA).

Methods

Pretreatment and radon-treatment of carbon paper-supported electrode: The pretreatment equipped with a classical three-electrode system containing the carbon paper-supported electrode as working electrode, a platinum wire as counter electrode, and an Ag/AgCl electrode as the reference electrode by applying a cycle potential from -2.0 V to +2.0 V for at least three times in PBS solution (pH 7.4). The electrode will be placed in the radon chamber for adsorption after washing and drying.

Characterize of carbon paper-supported electrode count rate: The treated carbon paper-supported electrodes were exposed in a radon chamber containing 2000 Bq/m^3 of radon gas for 6 h. Subsequently, the sample was placed in an HPGe gamma spectrometer and measured for 80,000 seconds to detect count rate at 295, 325 and 609 keV, after subtracting the environmental background. The experimental procedures adhere to China's national standard GB/T 11713-2015.

Scan-rate-dependent CV measurements of the radon-treated carbon paper-supported electrode: After radon adsorption onto the carbon paper-supported electrode, CV measurements were performed using a classical three-electrode system at scan rates of 0.02, 0.05, 0.10, 0.20, and 0.50 V/s under otherwise identical conditions.

Radon/xenon/krypton ECL measurement: The ECL system is equipped with a classical three-electrode system. The carbon paper-supported electrode as working electrode after radon and xenon treatment, a platinum wire as counter electrode, and an Ag/AgCl electrode as the reference electrode by applying a cycle potential from -2.0 V to +2.0 V in different solution.

Radon and xenon ECL spectrum measurement: ECL spectrum was measured by carbon paper-supported electrode as working electrode after radon and xenon treatment in different solution. Filters with 400, 428, 450, 500, 525, 536, 560, 580, 600, 630, 650 and 667 nm were applied to give the ECL intensity value at each wavelength.

EPR characterization of radon radical ions: Radon-containing gas with an activity concentration of 2000 Bq/m^3 was collected from the active area. Methanol containing 1% Bu_4NBF_4 and acetonitrile were separately purged with N_2 to remove dissolved O_2 and then saturated with the collected radon-containing gas. A radon-saturated carbon paper-supported electrode was secured with an electrode clip and subjected to 20 cyclic voltammetry (CV) cycles at a scan rate of 0.10 V/s. The anodic experiment was performed in the methanolic electrolyte over a potential range of 0 to +2.0 V, whereas the cathodic experiment was performed in the acetonitrile electrolyte over a potential range of 0 to -2.0 V. A continuous stream of radon-containing gas was maintained over the electrolyte surface throughout potential cycling. Immediately after the CV cycles, the electrolytes were analyzed by electron paramagnetic resonance (EPR) without delay.

HPLC-MS characterization of radon and xenon radical ions: For the radon experiments, radon-containing gas with an activity concentration of 2000 $Bq\ m^{-3}$ was collected from the active area. Methanol containing 1% Bu_4NBF_4 and acetonitrile containing 1% BF_3 were separately purged with N_2 to remove dissolved O_2 and were then saturated with the collected radon-containing gas. A radon-saturated carbon paper-supported electrode was secured with an electrode clip and subjected to 20 CV cycles at 0.10 $V\ s^{-1}$. The anodic experiment was performed in the methanolic electrolyte from 0 to +2.0 V, and the cathodic experiment was performed in the acetonitrile electrolyte from 0 to -2.0 V. A continuous stream of radon-containing gas was maintained over the electrolyte surface throughout potential cycling. Immediately after the CV cycles, the electrolytes were analyzed by high performance liquid chromatography-mass spectrometry (HPLC-MS) without delay. For the xenon experiments, the same procedure was followed using xenon-saturated methanolic and acetonitrile electrolytes, a

xenon-saturated carbon paper-supported electrode, and a continuous stream of xenon over the electrolyte surface. The electrolytes were analyzed by HPLC-MS immediately after potential cycling.

Calculation of LOD: The formula of LOD calculation: LOD = $3\sigma_{blank}/s$. In this formula, σ_{blank} is the standard deviation of blank carbon paper-supported electrodes and s is the slope value in Fig. 5B.

Computational method: The density functional theory (DFT) approach was used to fully optimize the geometries of the solvent molecules and radon. GAUSSIAN 09 [1] and 16 was used to compute the binding energy between solvent molecules and radon. The DFT optimization of the structure of C, N, H, O, S, Cl and Rn atoms was performed by wB97XD functional [2,3], for wB97XD is not only widely used for system with inter-molecule interactions as the exchange–correlation functional has long-range correction term [4], but also a long-range functional with dispersion-correction.[5,6] Def2svp basis set [7] is one of the few basis sets that can be used from hydrogen to radon and perform the best accuracy in the same level of computation. Based on def2TZVPP basis sets [8,9], which is used for the calculation of quantum chemical properties of radon oxides, Coupled Cluster [10,11] Singles Doubles and iterated Triples [CCSD(T)] energy calculations were obtained to demonstrate the binding of radon cation and anion radicals in solvents from the optimization procedure.

Classification algorithms and regularization strategies: To address the small-sample dataset, three classic machine learning algorithms with distinct principles were selected to construct the classifiers: logistic regression (LR), support vector machine (SVM),[12] and random forest (RF).[13] Given that small-sample data are highly susceptible to algorithmic overfitting and data leakage,[14] regularization techniques were deeply integrated into the model architectures. In the LR and SVM models, L2 regularization penalties were applied to constrain the norm of the weight vectors, thereby limiting model complexity and enhancing generalization capabilities on unseen data. For the ensemble-learning-based RF model, structural regularization was achieved by restricting the maximum depth of the decision trees (max_depth) and the minimum number of samples required to be at a leaf node (min_samples_leaf).[15] This effectively suppressed the individual trees from over-memorizing training set noise.

Correction of electrochemiluminescence data: Due to the long period of this experiment, PMT often does not give stable data for the same ECL intensity before and after a period of time at the same voltage. However, the ECL intensity for a certain concentration of $Ru(bpy)_3^{2+}$ can be considered consistent. Therefore, some of the ECL data covered in this manuscript used the method to correct the data as follows. A set of ECL intensities of $Ru(bpy)_3^{2+}$ was measured in supplement to the ECL signal test. When analyzing the data, the ECL signals of the carbon supported electrodes were corrected for equal proportions using the intensity of the $Ru(bpy)_3^{2+}$ ECL.

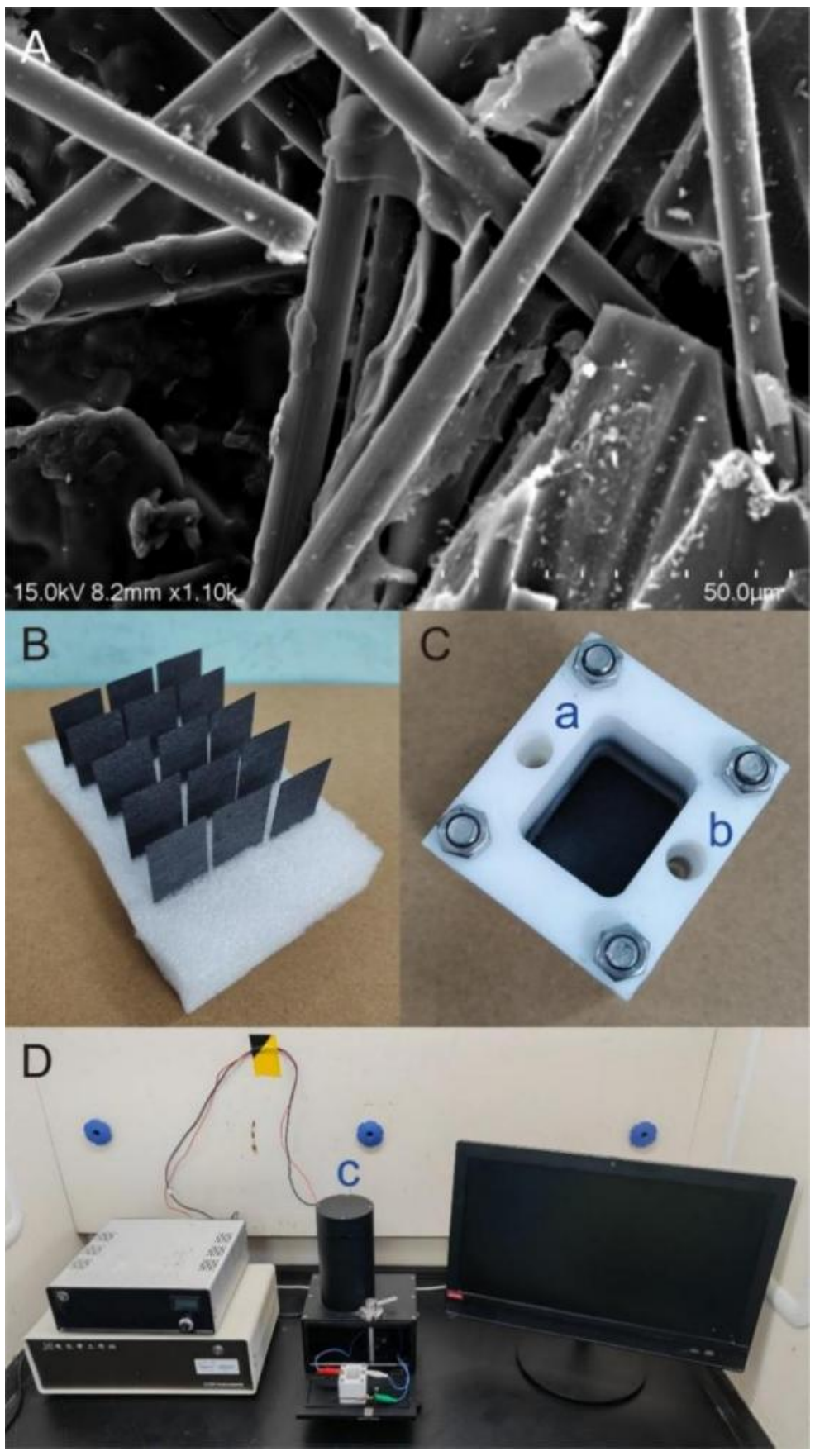

Fig. S1. Photographs of carbon paper-supported electrode and ECL device. (A) SEM image of carbon paper-supported electrode; (B) carbon paper-supported electrodes for radon adsorption; (C) electrolytic cell for detection of carbon paper-supported electrode; (D) ECL detection system containing controllers and an up-response ECL detector.

(The carbon paper-supported electrodes are placed in the radon chamber or practical environments for the radon adsorption as Fig. S1B. The radon adsorbed electrodes are installed in the electrolytic cell as working electrodes while the counter electrode (Pt) and the reference electrode (Ag/AgCl) are placed in the holes (a and b), respectively (Fig. S1C). According to Fig. S1D, the electrolytic cell is then applied to obtain the electrochemical and ECL data using the ECL system developed by our group with an up-sensing darkroom (c). The detailed information of this ECL system please see our previous study.[16])

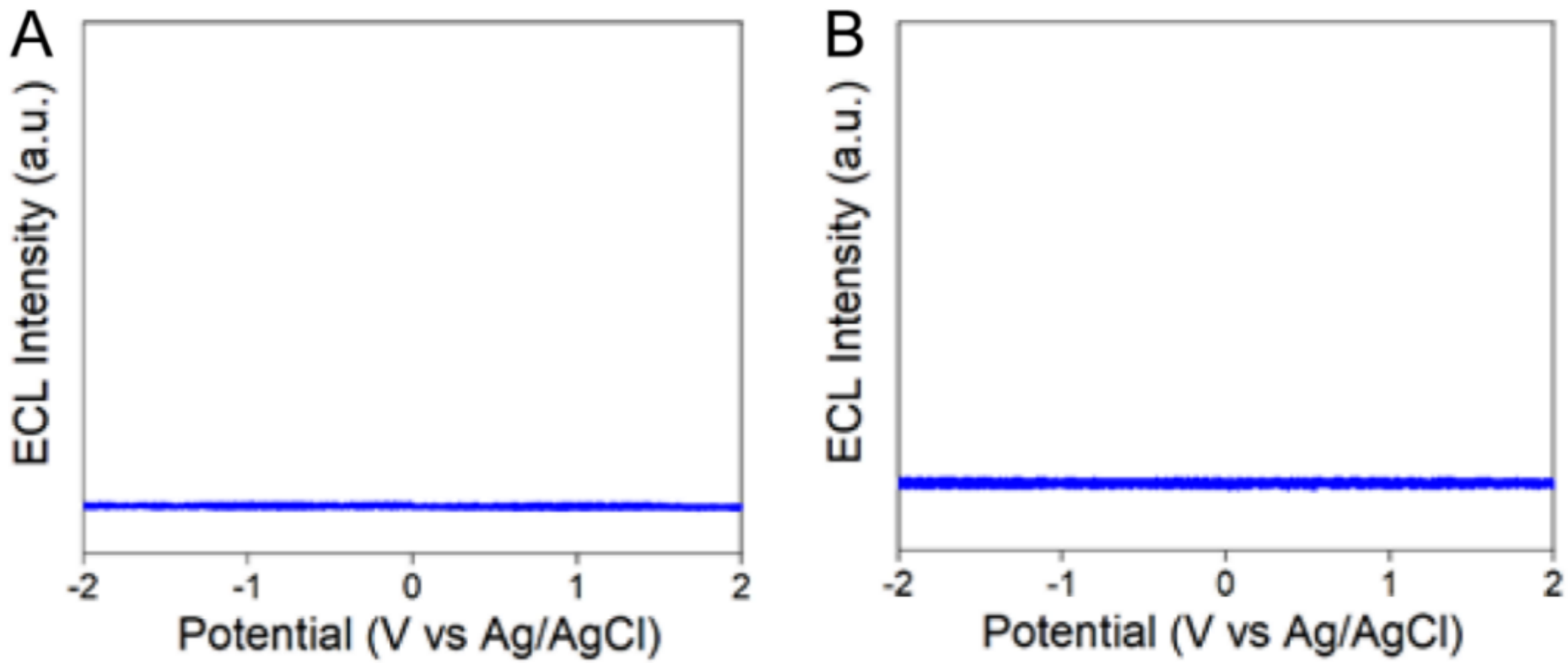

Fig. S2. ECL of carbon paper-supported electrode treated by Rn in PBS & CH_3CN. (A) ECL of carbon paper-supported electrode in 0.1 M pH 7.4 PBS solution. Scan rate: 100 mV/s, PMT: 900 V; (B) ECL of carbon paper-supported electrode in CH_3CN with 0.1 M Bu_4NBF_4. Scan rate: 100 mV/s, PMT: 900 V.

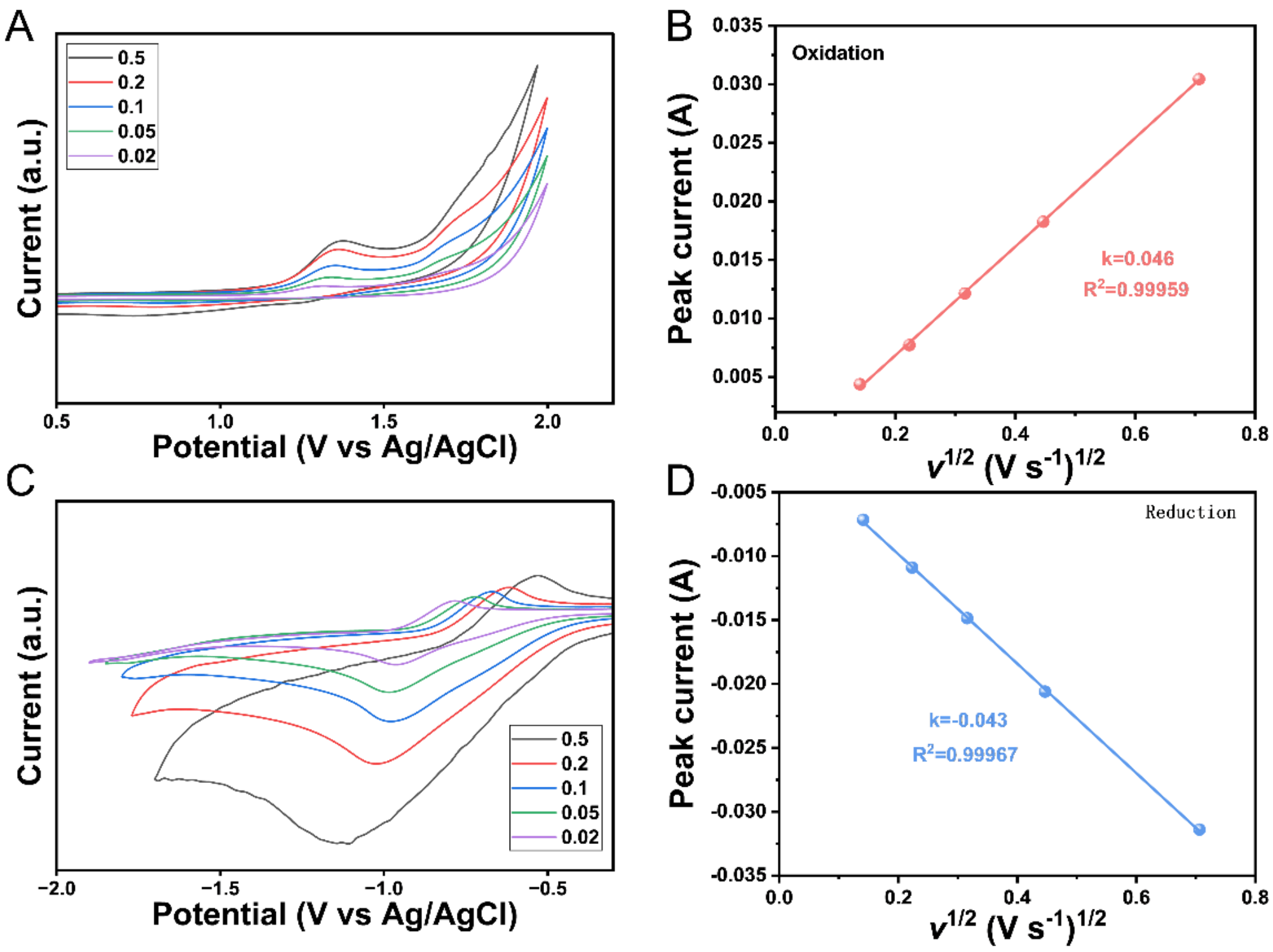

Fig. S3. Scan-rate-dependent electrochemical characterization of Rn-treated carbon paper-supported electrodes. (A) Anodic CV responses of the Rn (2000 Bq/m³)-treated carbon paper-supported electrode in 0.1 M PBS (pH 7.4) at scan rates of 0.02, 0.05, 0.10, 0.20 and 0.50 V/s. (B) Linear relationship between the anodic peak current (i_{pa}) and the square root of the scan rate ($v^{1/2}$). (C) Cathodic CV responses of the Rn (2000 Bq/m³)-treated carbon paper-supported electrode in CH_3CN containing 0.1 M Bu_4NBF_4 at the same scan rates. (D) Linear relationship between the cathodic peak current (i_{pc}) and $v^{1/2}$.

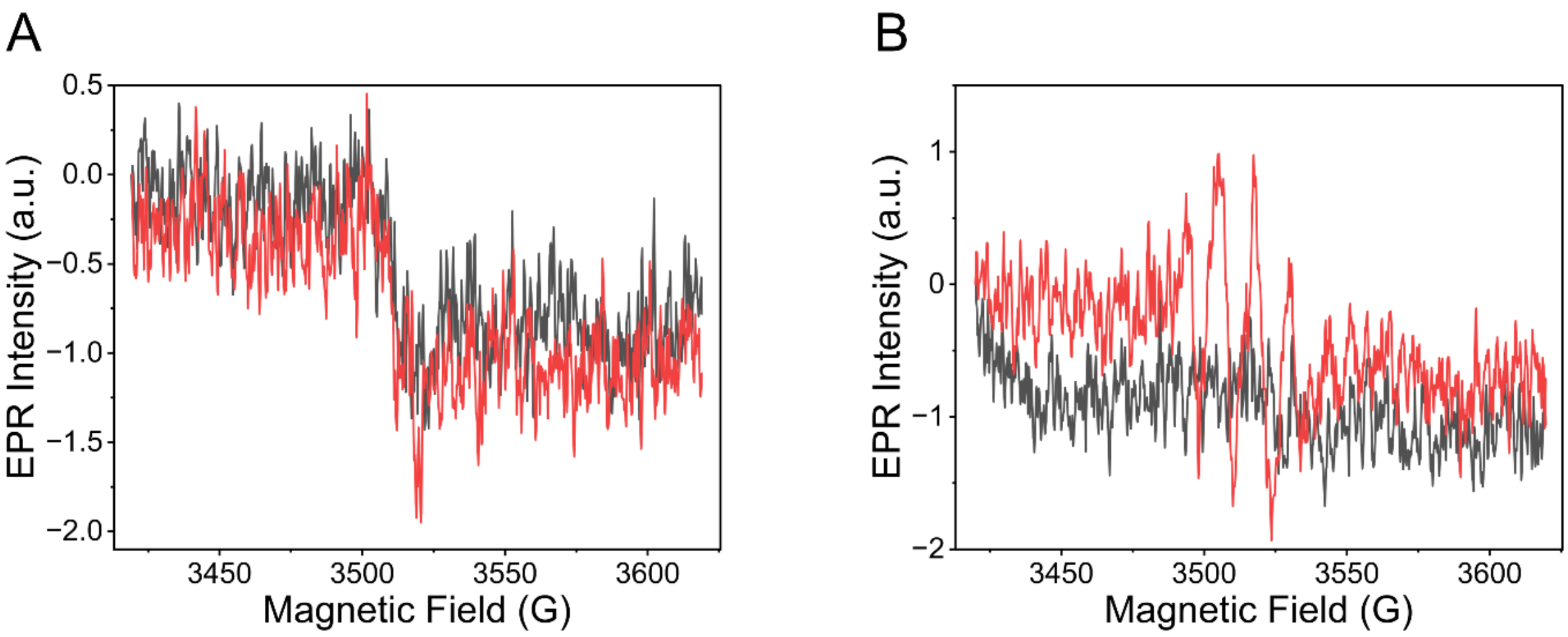

Fig. S4. EPR spectra supporting electrochemically generated Rn-associated radical species. (A) Representative anodic CW-EPR spectra of the Rn-containing methanolic electrolyte after electrolysis (red) and the matched Rn-free control (black). (B) Representative cathodic CW-EPR spectra of the Rn-containing acetonitrile electrolyte after electrolysis (red) and the matched Rn-free control (black).

(EPR can be used as corroborative evidence for the generation of radon in electrochemical reactions. The mean enhancement after correction for resonator Q and microwave power can be obtained as 37.7% in Fig. S4A, indicating the radical generation after electrochemical oxidation process. Under cathodic conditions, an additional radical response was also observed relative to the matched Rn-free control, supporting radical formation during the reduction process (Fig. S4B). The EPR results provide independent evidence for electrochemically generated Rn-associated radical intermediates.)

Program: Gaussian 16
Functional: PBE0
Basis and ECP: def2TZVP
D3 dispersion correction

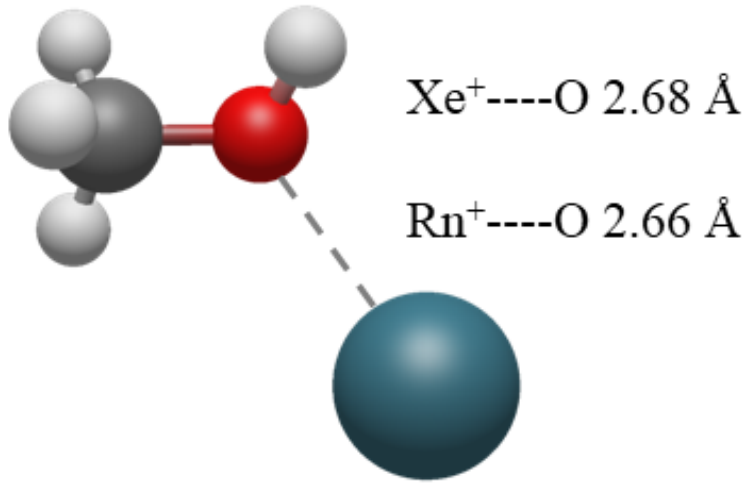

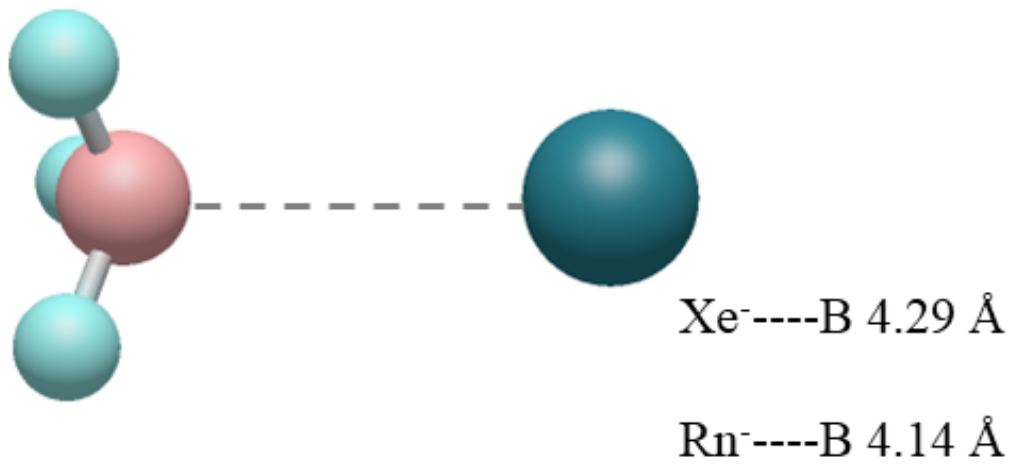

ions	KJ/mol	Kcal/mol
Xe^{+}	-236.5	-56.5
Rn^{+}	-196.5	-47.0

ions	KJ/mol	Kcal/mol
Xe^{-}	-713.3	-170.5
Rn^{-}	-555.1	-132.7

Adsorption energy = energy of combination – energy of ions – energy of CH_3OH/BF_3

Fig. S5. DFT-calculated stabilization of xenon and radon radical ions by CH_3OH and BF_3. Optimized structures, interaction distances, and adsorption energies of the CH_3OH complexes with $Xe^{\bullet+}$ and $Rn^{\bullet+}$ (left) and the BF_3 complexes with $Xe^{\bullet-}$ and $Rn^{\bullet-}$ (right).

(Calculations were performed using Gaussian 16 at the PBE0-D3/def2-TZVP level with the corresponding effective core potentials. The adsorption energies were calculated as $E_{ads} = E_{complex} - E_{ion} - E_{CH_3OH/BF_3}$. The negative adsorption energies indicate favorable stabilization of the noble-gas cations by CH_3OH and the corresponding anions by BF_3. DFT calculations showed favorable interactions of CH_3OH with $Xe^{\bullet+}$ and $Rn^{\bullet+}$, with adsorption energies as -236.5 and -196.5 kJ/mol, respectively. BF_3 exhibited even stronger stabilization of $Xe^{\bullet-}$ and $Rn^{\bullet-}$, with adsorption energies as −713.3 and −555.1 kJ/mol, respectively. These negative adsorption energies support the stabilization of the cationic species by CH_3OH and the anionic species by BF_3.)

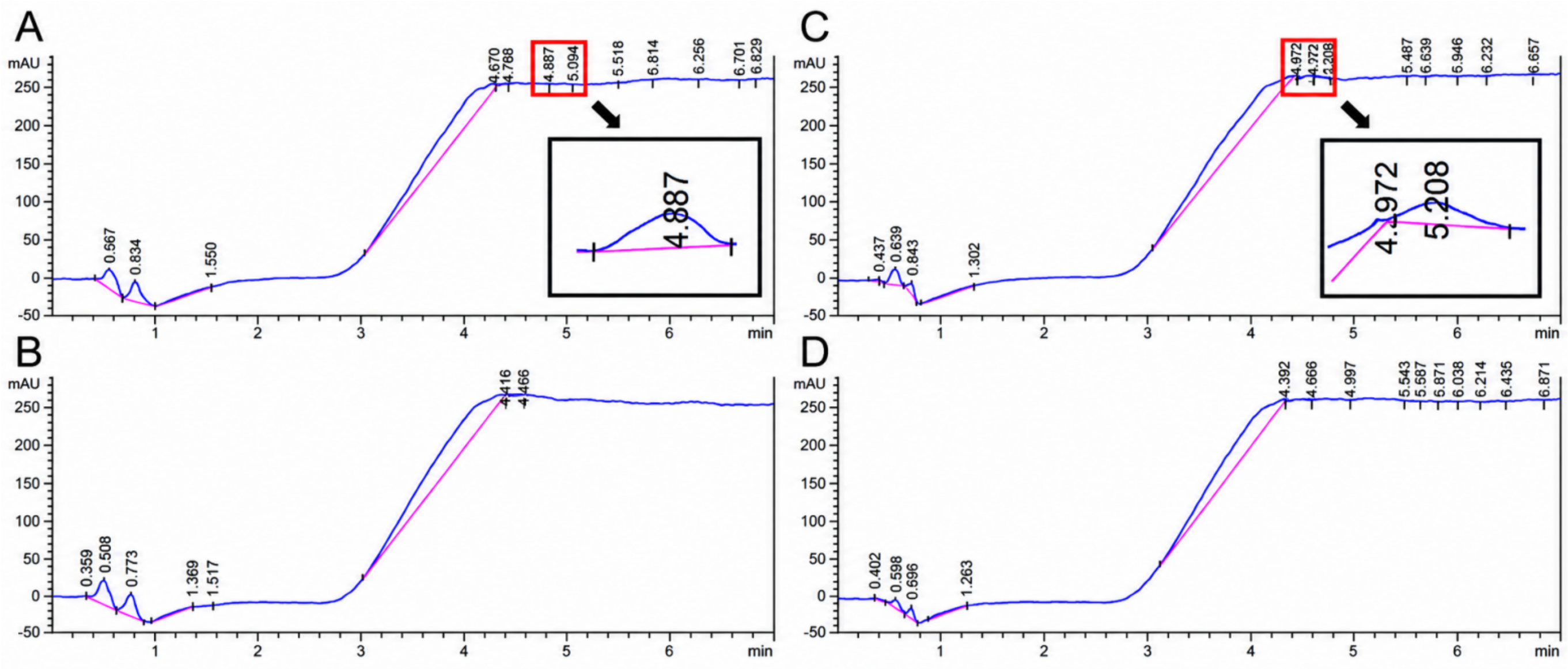

Fig. S6. HPLC chromatograms of the paper-supported electrode system. Anodic chromatograms of the methanolic electrolyte with (A) and without (B) exposure to radon at an activity concentration of 2000 Bq/m³. Cathodic chromatograms of the acetonitrile electrolyte with (C) and without (D) exposure to radon at 2000 Bq/m³.

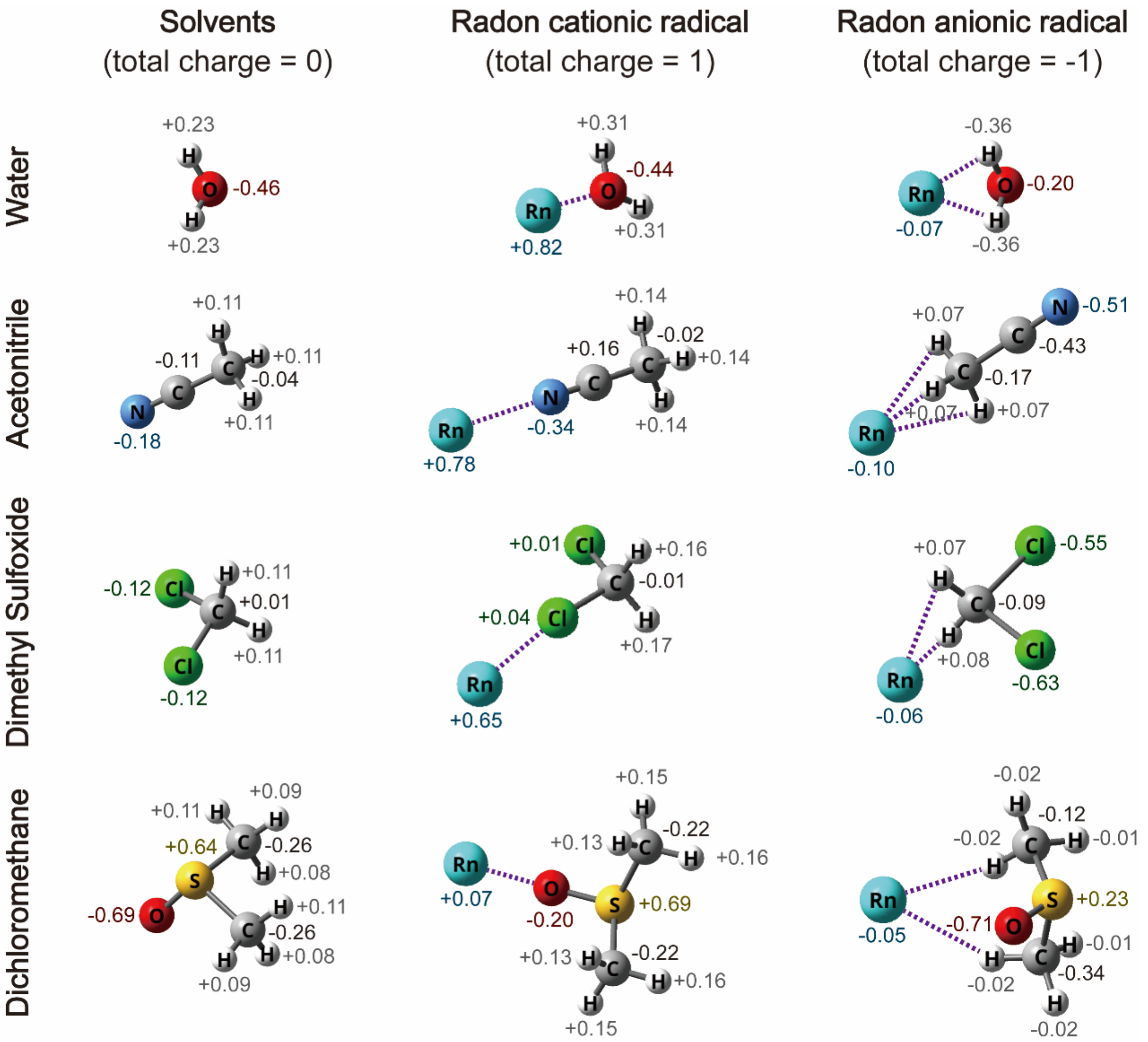

Fig. S7. Charge distribution *via* DFT calculations. Charge distribution of four solvent molecules binding with $Rn^{\bullet-}$/$Rn^{\bullet+}$ *via* DFT calculations.

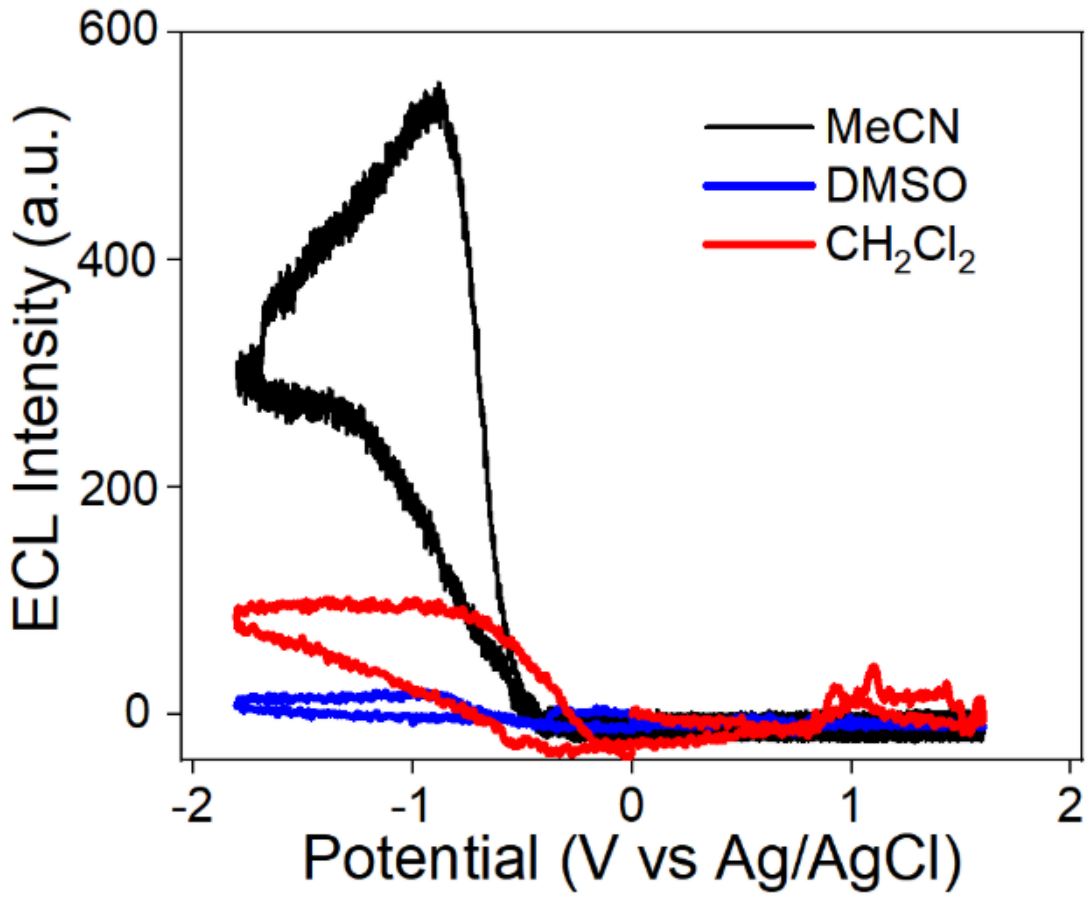

Fig. S8. ECL of carbon paper-supported electrode in organic solvents. ECL of radon (2000 Bq/m^3) treated carbon paper-supported electrode in different organic solvents with 0.1 M Bu_4NBF_4. Scan rate: 100 mV/s, PMT: 900 V.

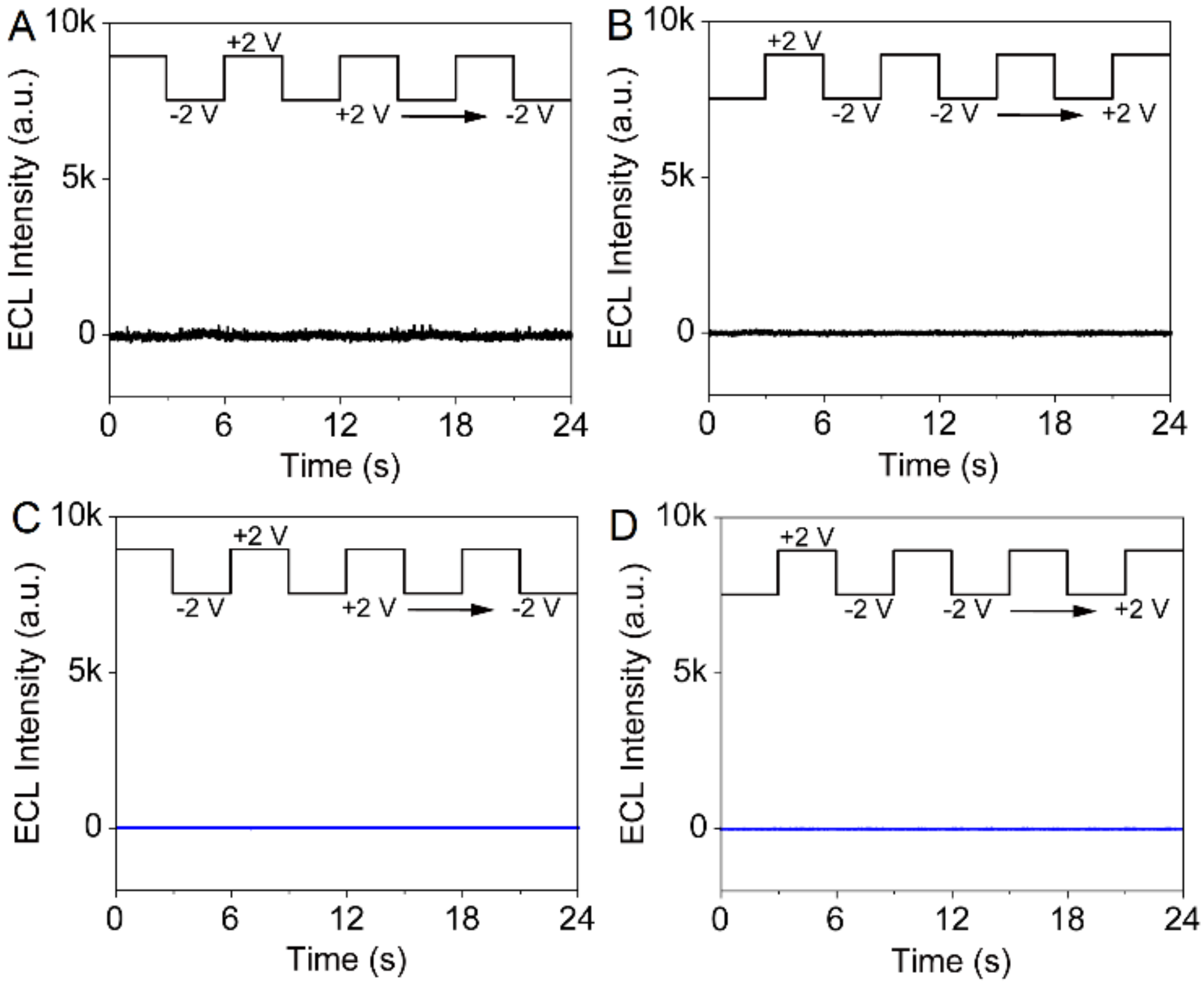

Fig. S9. ECL signals of blank electrode by CA method. ECL signals of blank electrode by CA method in 0.1 M PBS solution (black) and CH_3CN with 0.1 M Bu_4NBF_4 (blue) with the scan range from (A, C) +2 V to -2 V, (B, D) -2 V to +2 V. PMT: 900 V.

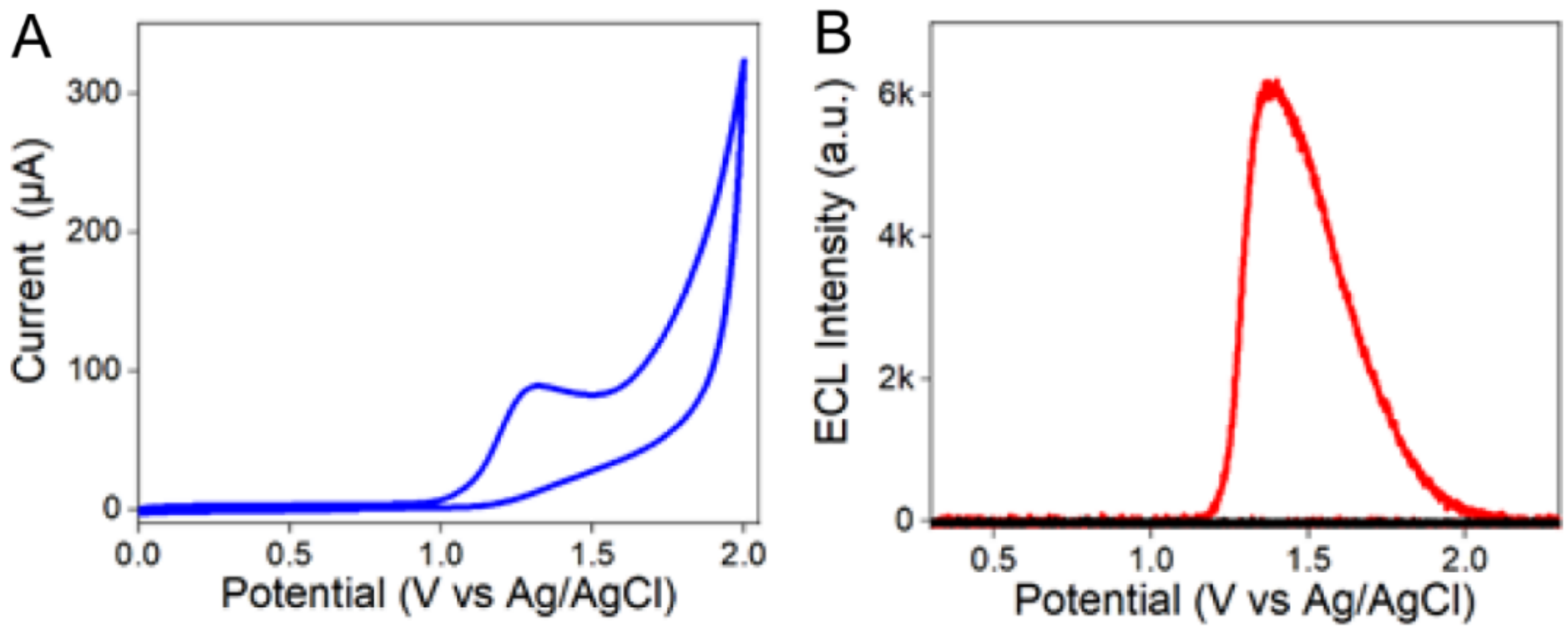

Fig. S10. Electrochemical analysis of aqueous solutions. (A) CV of 25 mM TPrA in 0.1 M pH 7.4 PBS solution; (B) ECL signal of carbon paper-supported electrode treated with (red) or without (black) 2000 Bq/m^3 radon in 0.1 M pH 7.4 PBS solution and 25 mM TPrA with (black) or without (red) 144 Bq/mL tritium water. Scan rate: 100 mV/s, PMT: 600 V.

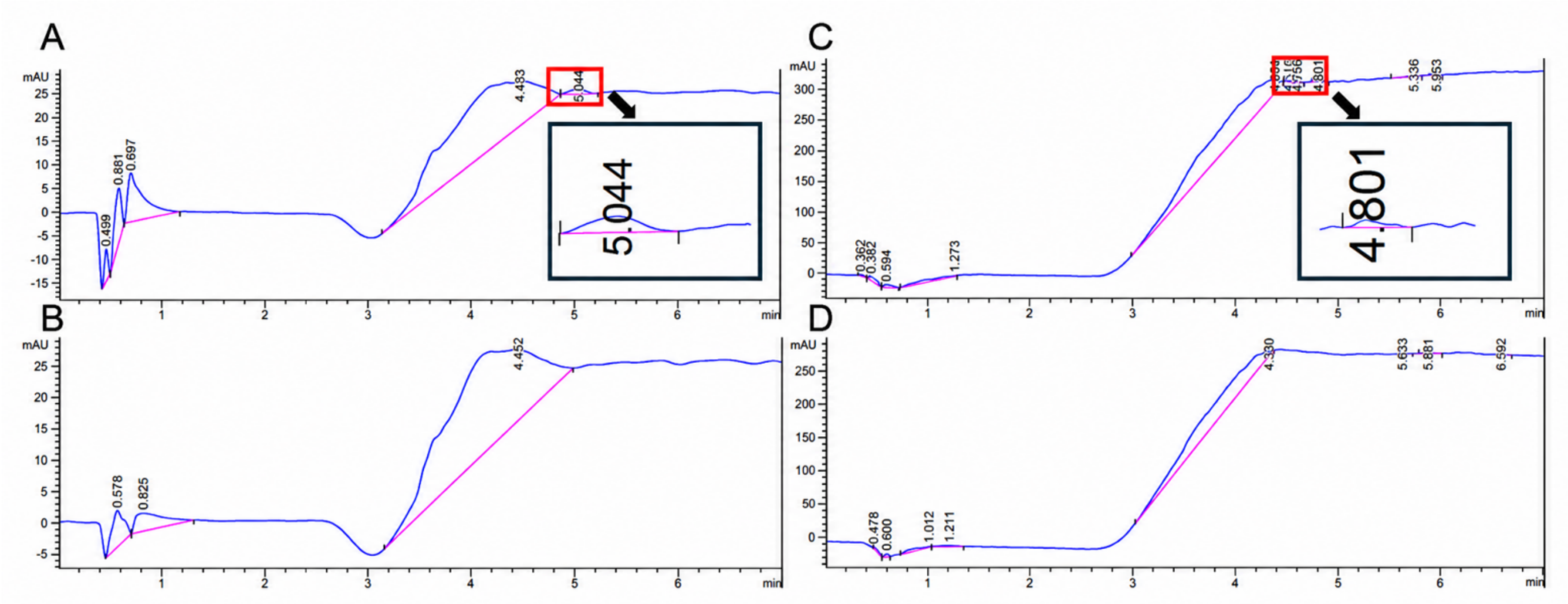

Fig. S11. HPLC chromatograms of the paper-supported electrode system. Anodic chromatograms of the xenon-saturated methanolic electrolyte (A) and the corresponding xenon-free control (B). Cathodic chromatograms of the xenon-saturated acetonitrile electrolyte (C) and the corresponding xenon-free control (D).

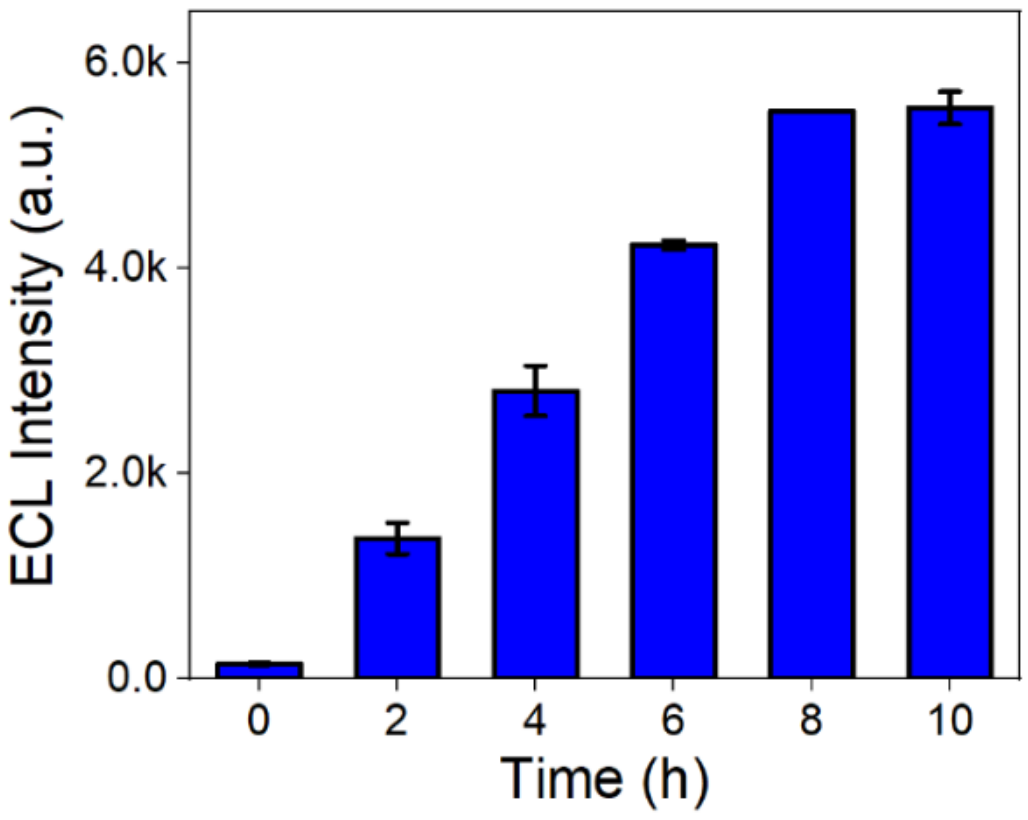

Fig. S12. ECL intensity of carbon paper-supported electrode treated for 0 to 10 h. ECL intensity of carbon paper-supported electrode treated with 2000 Bq/m^3 for 0 to 10 h in 0.1 M pH 7.4 PBS solution with 25 mM TPrA. Scan rate: 100 mV/s, PMT: 600 V.

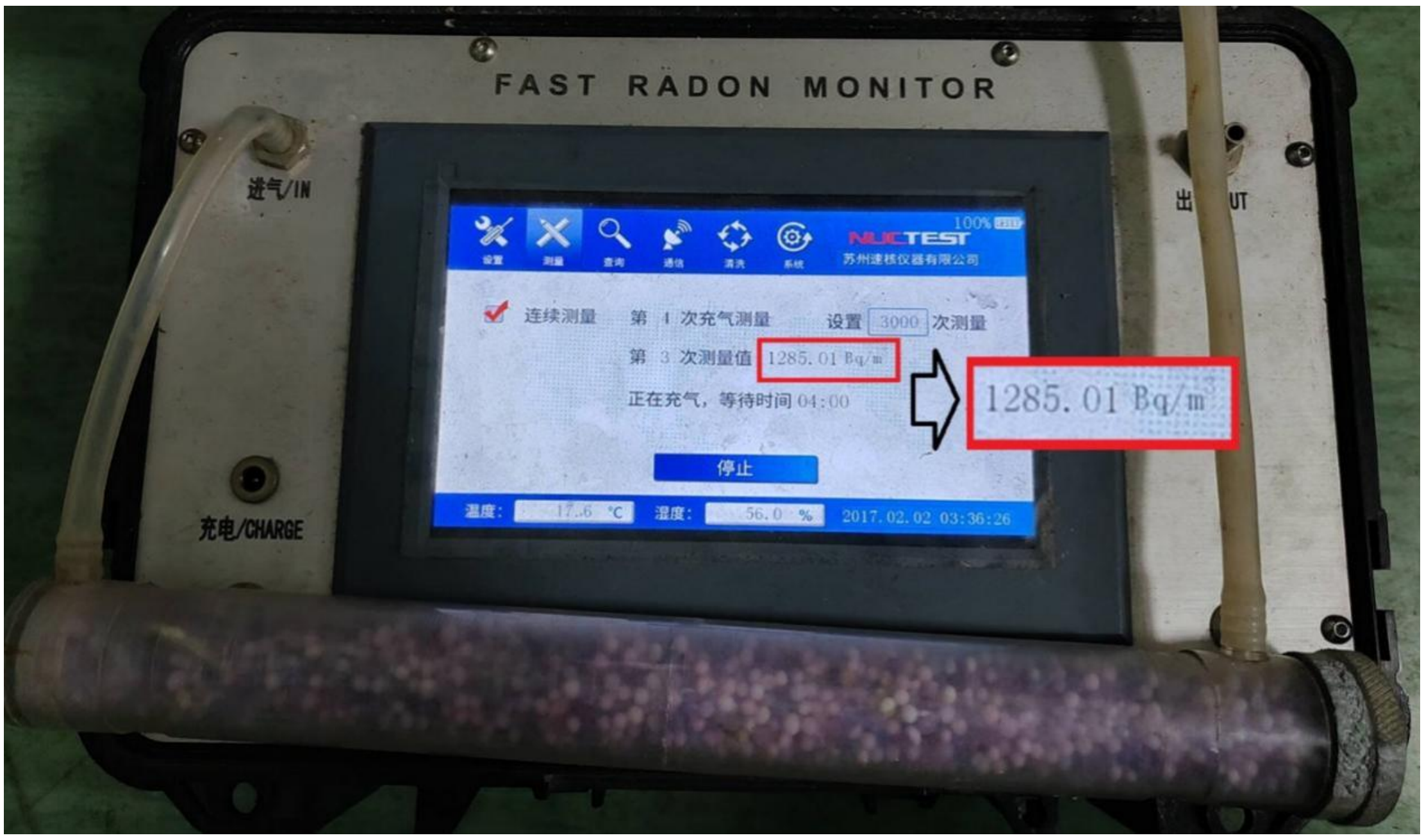

Fig. S13. Detection result of radon concentration in the basement. Detection result of radon concentration in the basement without good ventilation in Soochow University by using commercially available fast radon monitor.

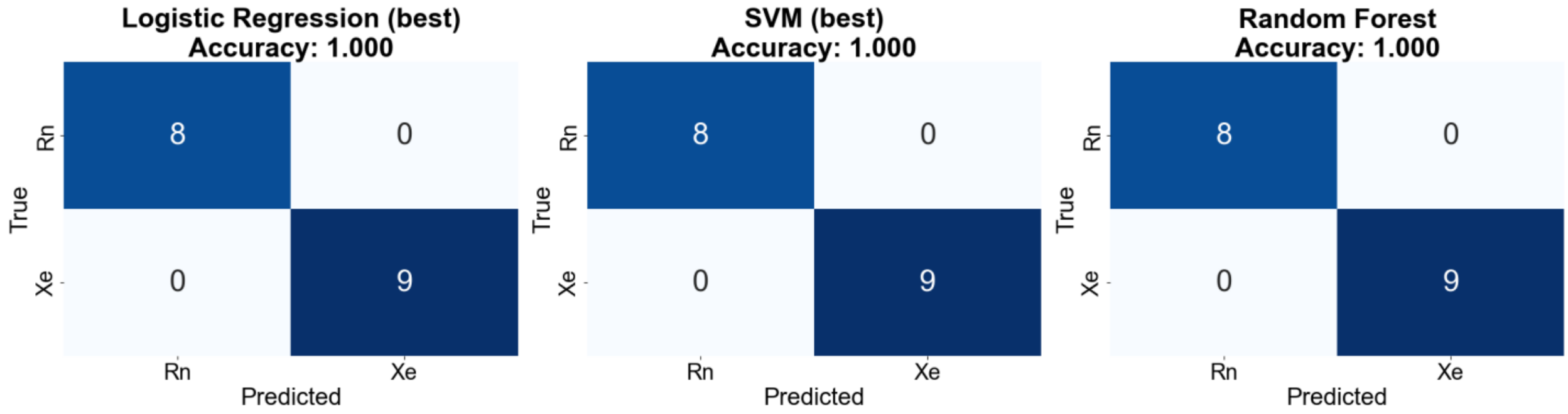

Fig. S14. Confusion matrices of the three machine learning classifiers. The matrices illustrate the exact match between the true experimental labels and the predicted labels for radon (Rn) and xenon (Xe) using LR, SVM, and RF models. The results demonstrate flawless 100% classification accuracy on the test set without any false classifications.

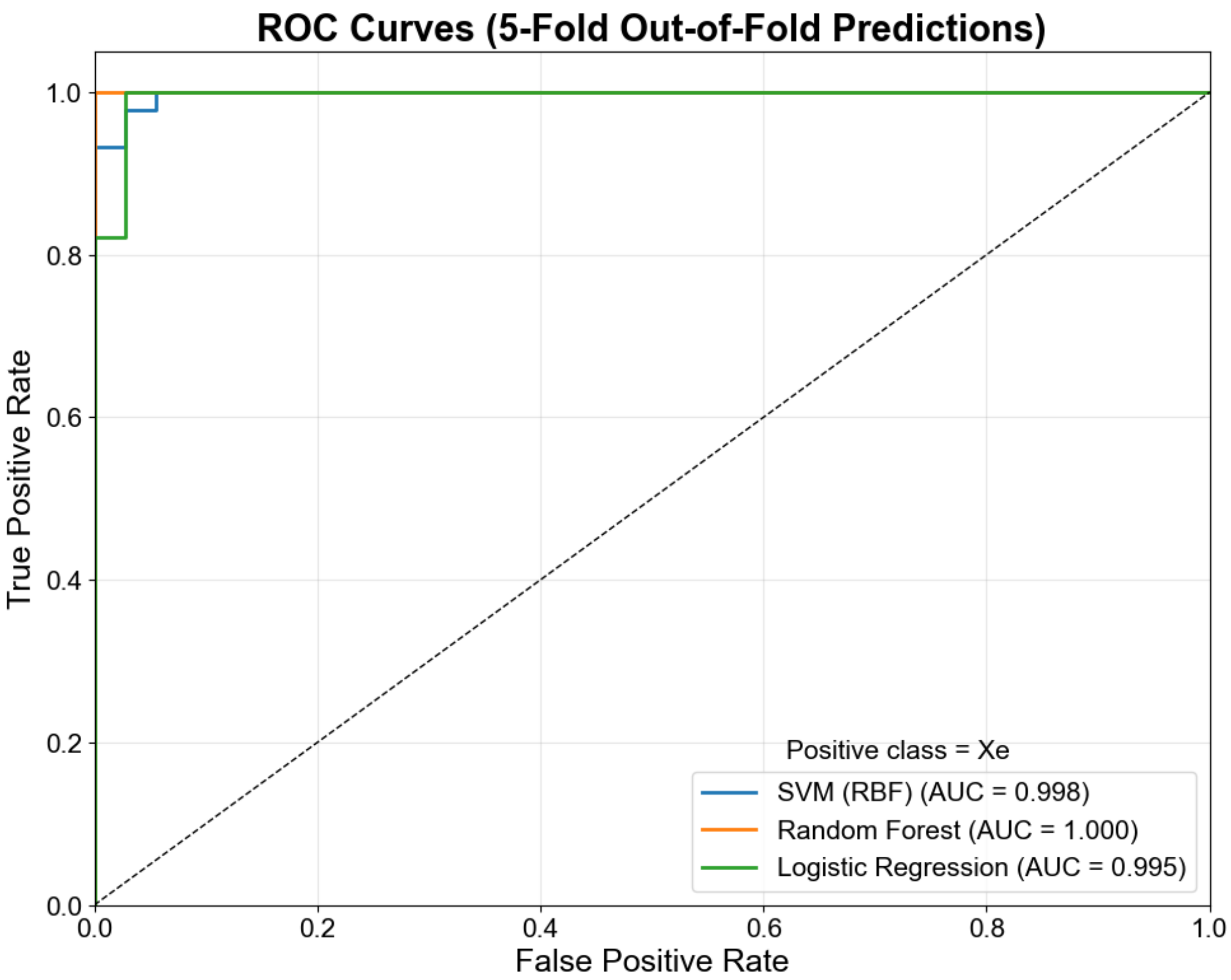

Fig. S15. Receiver operating characteristic (ROC) curves of the machine learning framework. The models were evaluated using a stratified 5-fold cross-validation approach to mitigate overfitting on the small-sample dataset. The exceptionally high Area Under the Curve (AUC) values demonstrate the robust discriminatory capability of the models in distinguishing between Rn and Xe ECL signals.

Table S1. First ionization energy for noble gases.

Nobel Gas	Rn	Xe	Kr	Ar	Ne	He
First Ionization Energy (kJ/mol)	1037.1	1170.4	1350.8	1520.6	2080.7	2372.3

NIST Atomic Spectra Database (ASD): * Kramida, A., Ralchenko, Yu., Reader, J., and NIST ASD Team (2024). *NIST Atomic Spectra Database (version 5.11)*. Available at: https://physics.nist.gov/asd.

Table S2. The LODs of Rn detection methods in standards and previous literature.

	Rn detection methods	LODs	Ref.
Alpha-spectrometry detection (WHO Handbook on indoor radon: a public health perspective)	Alpha-track detector	30 Bq/m^3	--
	Active charcoal adsorption detector	20 Bq/m^3	--
	Electronic integrating device	20 Bq/m^3	--
	Continuous Rn monitor (pump used)	5 Bq/m^3	--
Alpha-spectrometry detection (HJ 1212-2021, National Eco-Environmental Standard of P. R. China)	Track etching method	5 Bq/m^3	--
	Active carbon box method	6 Bq/m^3	--
	Pulse ionization chamber method	5 Bq/m^3	--
	Electrostatic collection method	5 Bq/m^3	--
Chemical probes	Malachite green fluorescence probe	2060 $Bq\ h/m^3$	[17]
	GR5 DNAzyme-based colorimetric detection	186.48 $Bq\ h/m^3$	[18]
	PS2.M ssDNA based colorimetric detection	1960 $Bq\ h/m^3$	[19]
	OliGreen based fluorescence probe	1963 $Bq\ h/m^3$	[20]
	Duplex-hemin/G-quadruplex based colorimetric detection	1620 $Bq\ h/m^3$	[21]
ECL sensors	Pb-210 based ECL sensor	0.9 Bq/m^3	[22]
	This work	8 mBq/m^3	--

Notes and references

1 M. J. Frisch, G. W. Trucks, H. B. Schlegel, G. E. Scuseria, M. A. Robb, J. R. Cheeseman, G. Scalmani, V. Barone, G. A. Petersson, H. Nakatsuji, X. Li, M. Caricato, A. Marenich, J. Bloino, B. G. Janesko, R. Gomperts, B. Mennucci, H. P. Hratchian, J. V. Ortiz, A. F. Izmaylov, J. L. Sonnenberg, D. Williams-Young, F. Ding, F. Lipparini, F. Egidi, J. Goings, B. Peng, A. Petrone, T. Henderson, D. Ranasinghe, V. G. Zakrzewski, J. Gao, N. Rega, G. Zheng, W. Liang, M. Hada, M. Ehara, K. Toyota, R. Fukuda, J. Hasegawa, M. Ishida, T. Nakajima, Y. Honda, O. Kitao, H. Nakai, T. Vreven, K. Throssell, J. A. Montgomery, Jr., J. E. Peralta, F. Ogliaro, M. Bearpark, J. J. Heyd, E. Brothers, K. N. Kudin, V. N. Staroverov, T. Keith, R. Kobayashi, J. Normand, K. Raghavachari, A. Rendell, J. C. Burant, S. S. Iyengar, J. Tomasi, M. Cossi, J. M. Millam, M. Klene, C. Adamo, R. Cammi, J. W. Ochterski, R. L. Martin, K. Morokuma, O. Farkas, J. B. Foresman and D. J. Fox, Gaussian 09, revision D.01, Gaussian, Inc., Wallingford, CT, 2016.

2 S. Grimme, *J. Comput. Chem.*, 2006, **27**, 1787.

3 J.-D. Chai and M. Head-Gordon, *J. Chem. Phys.*, 2008, **128**, 084106.

4 N. Yamada, Y. Shin, K. Kawasaki, A. Yokoyama and T. Ida, *J. Radioanal. Nucl. Chem.*, 2018, **318**, 2303.

5 M. Lalitha and S. Lakshmipathi, *Phys. Chem. Chem. Phys.*, 2017, **19**, 30895.

6 Y. Lin, G. Li, S. Mao and J. Chai, *J. Chem. Theory Comput.*, 2013, **9**, 263.

7 F. Weigend, F. Furche and R. Ahlrichs, *J. Chem. Phys.*, 2003, **119**, 12753.

8 F. Weigend and R. Ahlrichs, *Phys. Chem. Chem. Phys.*, 2005, **7**, 3297.

9 N. A. G. Bandeira and J. Marçalo, *Phys. Chem. Chem. Phys.*, 2023, **25**, 14084.

10 R. J. Bartlett and G. D. Purvis, *Int. J. Quantum Chem.*, 1978, **14**, 561.

11 R. J. Bartlett, *Annu. Rev. Phys. Chem.*, 1981, **32**, 359.

12 C. Cortes and V. Vapnik, *Mach. Learn.*, 1995, **20**, 273.

13 L. Breiman, *Mach. Learn.*, 2001, **45**, 5.

14 N. Artrith, A. Urban, Y. Wang and G. Ceder, *Nat. Chem.*, 2021, **13**, 505.

15 F. Pedregosa, G. Varoquaux, A. Gramfort, V. Michel, B. Thirion, O. Grisel, M. Blondel, P. Prettenhofer, R. Weiss, V. Dubourg and J. VanderPlas, *J. Mach. Learn. Res.*, 2011, **12**, 2825.

16 Z. Wang, L. Fang, J. Zhao, C. Li, H. Xie, J. B. Pan and D. Hua, *Adv. Sci.*, 2026, **13**, e06325.

17 S. Li, H. Liu, G. Yang, S. Liu, R. Liu and C. Lv, *J. Environ. Radioact.*, 2018, **195**, 60.

18 H. Liu, Y. Chen, C. Song, G. Tian, S. Li, G. Yang and C. Lv, *Anal. Bioanal. Chem.*, 2018, **410**, 4227.

19 H. Liu, Y. Ji, C. Song, G. Tian, S. Li, G. Yang and C. Lyu, *Chin. J. Anal. Chem.*, 2018, **46**, 658.

20 M. Long, H. Deng, G. Tian, C. Song, H. Liu, Y. Shen and C. Lv, *Anal. Chim. Acta*, 2016, **936**, 202.

21 G. Yang, C. Song, Q. Shi, H. Liu, S. Li, R. Liu, S. Liu and C. Lv, *J. Pharm. Biomed. Anal.*, 2018, **159**, 459.

22 Z. Wang, Y. Li, J. B. Pan, M. Xu, J.-J. Xu and D. Hua, *J. Hazard. Mater.*, 2023, **453**, 131449.
